# Plant hydraulic traits reveal islands as refugia from worsening drought

**DOI:** 10.1093/conphys/coz115

**Published:** 2020-01-29

**Authors:** Aaron R Ramirez, Mark E De Guzman, Todd E Dawson, David D Ackerly

**Affiliations:** 1 Department of Integrative Biology, University of California, 3040 Valley Life Sciences Building #3140 Berkeley CA 94720-3200, USA; 2 Department of Biology & Environmental Studies, Reed College, Portland, 33203 Southeast Woodstock Blvd., Portland, Oregon 97202-8199, USA; 3 Department of Botany & Plant Sciences, University of California, Riverside, 900 University Ave., Riverside CA 92521, USA; 4 Department of Environmental Science, Policy, and Management, University of California, 130 Mulford Hall #3114, Berkeley, CA 94720-3114, USA

**Keywords:** chaparral, climate change, climate refugia, drought, hydraulic safety margins, island ecosystems

## Abstract

Relatively mesic environments within arid regions may be important conservation targets as ‘climate change refugia’ for species persistence in the face of worsening drought conditions. Semi-arid southern California and the relatively mesic environments of California’s Channel Islands provide a model system for examining drought responses of plants in potential climate change refugia. Most methods for detecting refugia are focused on ‘exposure’ of organisms to certain abiotic conditions, which fail to assess how local adaptation or acclimation of plant traits (i.e. ‘sensitivity’) contribute to or offset the benefits of reduced exposure. Here, we use a comparative plant hydraulics approach to characterize the vulnerability of plants to drought, providing a framework for identifying the locations and trait patterns that underlie functioning climate change refugia. Seasonal water relations, xylem hydraulic traits and remotely sensed vegetation indices of matched island and mainland field sites were used to compare the response of native plants from contrasting island and mainland sites to hotter droughts in the early 21st century. Island plants experienced more favorable water relations and resilience to recent drought. However, island plants displayed low plasticity/adaptation of hydraulic traits to local conditions, which indicates that relatively conserved traits of island plants underlie greater hydraulic safety and localized buffering from regional drought conditions. Our results provide an explanation for how California’s Channel Islands function as a regional climate refugia during past and current climate change and demonstrate a physiology-based approach for detecting potential climate change refugia in other systems.

## Introduction

Plants in the 21st century are exposed to hotter and more frequent climate change-driven drought conditions (Breshears *et al.,* 2005; Seager*,* 2007; Williams *et al.,* 2013; Trenberth *et al.,* 2014; Allen *et al.,* 2015). The persistence of species that experience such abiotic conditions may depend, in part, on the presence of relatively mesic environments within drought-exposed landscapes—i.e. ‘hydrologic refugia’ (McLaughlin *et al.,* 2017; Cartwright, 2018). The detection and protection of such climate change refugia is a key strategy for species conservation (Keppel *et al.,* 2011; Morelli *et al.,* 2016). However, existing methods for detecting refugia that involve modeling the persistence of abiotic conditions within the suitable range for target species or ecosystems (e.g. bioclimate envelope models) do not provide direct information on the *sensitivity* of target species due to local adaptation or acclimation (Pearson and Dawson, 2003). Assessing both the extent to which species are exposed to climate change driven droughts and how local adaptation and acclimation contribute to species sensitivity is necessary for accurately predicting the responses of ecosystems to future conditions (Williams *et al.*, 2008a; Crausbay *et al.* 2017).

**Figure 1:**
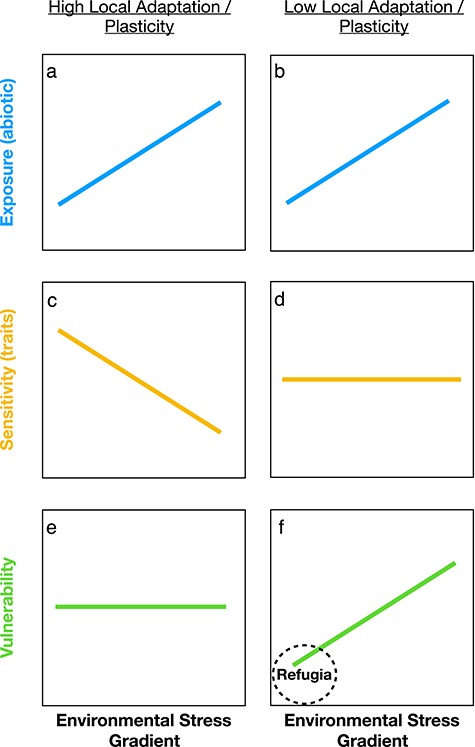
Conceptual figure depicting two hypothesized relationships between exposure (**a** and **b**), sensitivity (**c** and **d**) and vulnerability (**e** and **f**) that depend on how plant physiological traits vary across gradients. On one hand, if sensitivity is determined by traits that are fine-tuned to local conditions (c)—either through local adaptation or phenotypic plasticity—then there is likely to be static vulnerability across the gradient (e). However, if sensitivity is determined by traits that are relatively conserved across a stress gradient (d), then vulnerability will be reduced in low exposure environments (right).

Frameworks for identifying and assessing conservation targets that are based on a more complete understanding of plant physiological responses to environmental stress are needed to address the threats climate change poses to ecosystems (Madlinger *et al*., 2018). Comparative trait-based approaches may be able to improve detection of climate change refugia by directly assessing the true vulnerability (exposure + sensitivity) of plants to increasing drought (Fig. 1). Of particular utility in this context is the characterization of traits associated with plant hydraulic function and carbon gain, which can be predictive of drought-induced plant mortality under a warmer, drier future climate (McDowell *et al.,* 2008; Anderegg *et al.*, 2016; Venturas *et al.,* 2017; Choat *et al.,* 2018). While there may be multiple, interacting mechanisms that drive plant mortality during drought (Sala *et al.,* 2010;Jacobsen *et al.,* 2011; Sevanto *et al.,* 2014), the risk of hydraulic failure—i.e. the loss of the ability to transport water due to drought-induced xylem cavitation—is predictive across a wide range of species and ecosystems (Jacobsen *et al.,* 2007b; Choat *et al.,* 2012, 2018; Anderegg *et al.,* 2016; Adams *et al.,* 2017). Measurements of hydraulic traits can be combined with seasonal water relations to estimate ‘hydraulic safety margins’ as the difference between minimum water status (Ψ_min_) and the xylem pressure potential causing significant xylem dysfunction (e.g. P_50_). Hydraulic safety margins provide important, comparable information on the hydraulic function of plants experiencing contrasting environmental conditions that can be predictive of future drought-induced mortality (Choat *et al.,* 2012; Skelton *et al.,* 2015; Anderegg *et al.,* 2016).

Islands are generally cooler, wetter and less seasonal than mainland environments due to strong maritime influences on island climate (Weigelt *et al.,* 2013). Such maritime conditions within semi-arid regions like southern California involve increased coastal fog occurrence that may locally buffer plants from exposure to severe drought conditions (Fischer *et al.,* 2009; Vasey *et al.,* 2012; McLaughlin *et al.,* 2017). In addition, modeling approaches suggest that areas with strong coastal influence will experience less pronounced warming over the next century (Lebassi *et al*., 2009; Potter, 2014). These factors may allow islands to function as regional climate refugia during worsening drought conditions with climate change. However, whether local adaptation and/or phenotypic plasticity of plant hydraulic traits can offset the benefits of more mesic environments is poorly understood. Comparing hydraulic traits and, in particular, hydraulic safety margins of island and mainland plant communities would provide a test of relative drought vulnerability in these contrasting environments.

Southern California and the adjacent California Channel Islands present an ideal study system for evaluating hydraulic safety in island versus mainland environments. During seasonal and inter-annual droughts in this region, mature chaparral shrubs can experience significant dieback and mortality (Schlesinger and Gill, 1978; Schlesinger *et al.,* 1982; Davis *et al.,* 2002, Paddock *et al.,* 2013, Pratt *et al.,* 2014, Venturas *et al.,* 2016). As a result, these environments have selected for plants with highly drought tolerant functional strategies, including many with high xylem cavitation resistance (Ackerly, 2004; Bhaskar *et al.,* 2007; Jacobsen *et al.,* 2008). Previous studies in these systems have shown that cavitation resistance and hydraulic safety can vary seasonally (Jacobsen *et al.,* 2007a, 2014; Pivovaroff *et al.,* 2015) and inter-annually (Jacobsen *et al.,* 2007b) within particular species. Also, hydraulic traits have been shown to vary between species with different life history strategies (Redtfeldt and Davis, 1996; Kolb and Davis, 1994; Davis *et al.,* 1999; Jacobsen *et al.,* 2007b) and across different semi-arid plant communities (i.e. chaparral, coastal sage scrub, and desert scrub) in southern California (Jacobsen *et al.,* 2008). However, few studies have investigated hydraulic traits on the California Channel Islands (But, see Jacobsen *et al*., 2018), where the maritime climate and geographic isolation of island environments create a high likelihood of evolutionary divergence in adaptive traits (Ackerly, 2003).

In addition, biogeographic patterns of vegetation on the California Channel Islands have long been suspected as evidence of more buffered conditions on the islands that allowed the persistence of taxa once widespread in other parts of California (Axelrod, 1967; Raven and Axelrod, 1978). The existing narrative for this pattern is that the islands provided a cooler, wetter climate that facilitated this persistence. A trait-based approach to testing the hypothesis that the islands function as a climate, and more specifically a hydrologic, refugia could provide a mechanistic explanation for these long-observed biogeographic patterns.

A common limitation of studies that compare traits across environments is not accounting for phylogenetic relationships between taxa, which can make it difficult to interpret trait patterns driven by the statistical non-independence of closely related species (Felsenstein, 1985; Harvey and Pagel, 1991; Maherali *et al.,* 2004). One solution to this problem is to incorporate phylogenetic information that can be used to investigate differences among a series of closely related taxonomic pairs (Westoby *et al.,* 1998; Westoby, 1999; Ackerly, 2000). Collecting data that takes into account the phylogenetic relationships between taxa allows for more accurate linkage of trait patterns to the underlying responses to different environments (Ackerly and Donoghue, 1998).

**Figure 2:**
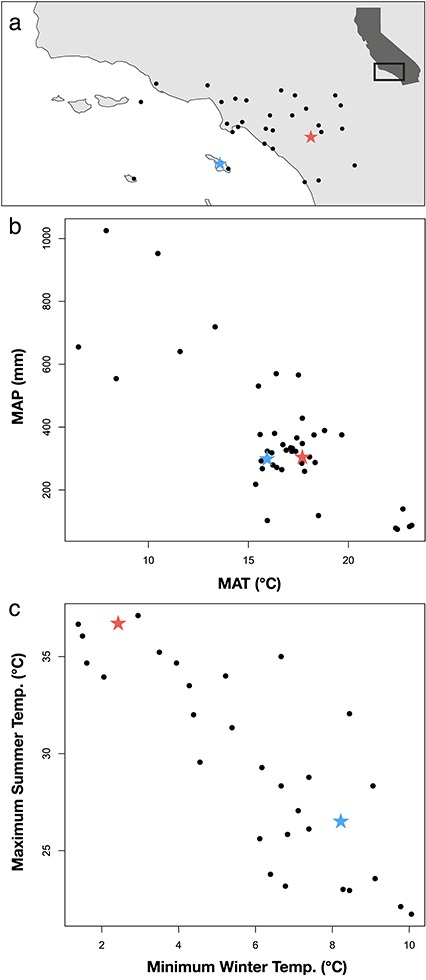
Locations (**a**), mean annual climate (**b**) and temperature seasonality (**c**) of southern California region based on historical weather station records (*via* Western Regional Climate Center: http://www.wrcc.dri.edu). Each closed circle represents climate means from a single weather station. Matched island (Santa Catalina Island) and mainland (Santa Ana Mountains) field sites are indicated by a blue and red star, respectively, in each figure. Despite their proximity and similar mean annual climates, island and mainland field sites are on opposite ends of the temperature seasonality spectrum, with the island site experiencing lower summer and higher winter temperatures (i.e. a more maritime climate). Inset in Fig. 4a shows the study region within the state of California.

**Figure 3:**
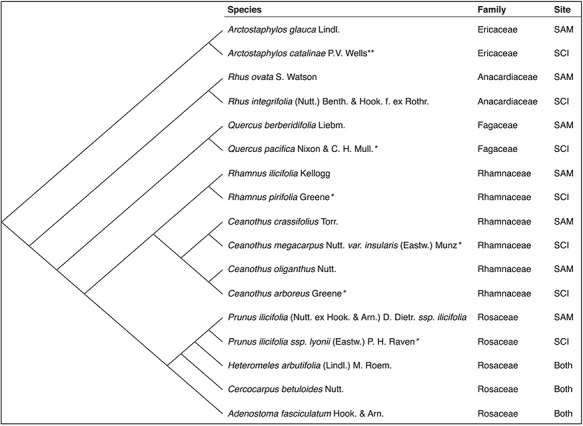
Phylogenetic relationships between the 20 species (10 species pairs) included in the study. Relationships are based on a recent phylogenetic supertree (R2G2_20140601; *available online*). The tree was constructed using the software program phylomatic. Seven of the species pairs represent congeneric pairs, i.e. one species occurring on the mainland and the other on the island. The remaining three pairs are conspecific pairs, where different populations of the same species occur at each site. Single asterisk denotes a species endemic to the CA Channel Islands; double asterisks denote a species endemic to Santa Catalina Island.

Here, we present data to assess the relative drought vulnerability of native woody plant communities on the California Channel Islands and the adjacent southern California mainland, sites which harbor similar plant assemblages but differ in the strength of maritime-influence on the local climate (Figs 2 and 3). Our research aims to (i) determine if the maritime climate of island environments is sufficient to buffer native California plants from 21st century drought conditions exacerbated by climate change and (ii) to evaluate a trait-based method for detecting the physiological mechanisms of hydrologic refugia that can be applied to other systems.

## Materials and methods

### Study sites and species

Matched island and mainland sites were used to compare the seasonal water relations and cavitation resistance of chaparral shrubs on Santa Catalina Island (SCI) and the adjacent southern California mainland. The island site was located on the east (channel-facing) slope of SCI near Blackjack Mountain (33°23′38.1″N; 118°23′50.4″W). The mainland site was located on the east slope of the Santa Ana Mountains in the Cleveland National Forest (33°38′44.6″N; 117°23′46.6″W), overlooking Lake Elsinore, CA (Fig. 2). These sites were selected to maximize similarity in latitude, slope, aspect, elevation, soil characteristics, mean annual precipitation/temperature and species composition across the island–mainland environments (Table 1, Fig. 2).

**Table 1:** Summary of site characteristics for matched island and mainland field sites

Site	Aspect	Elevation	Total N (%)	Total C (%)	Sand/silt/clay (%)
Catalina Island	NE	500 m	0.160 ± 0.042	3.037 ± 1.122	62/24/14
Santa Ana Mountains	NE	900 m	0.157 ± 0.046	2.398 ± 0.77.	66/21/13

Soil measurements of Total *N*, Total *C* and particle size (% sand, % silt and % clay) are based on 0–10 cm soil samples. Soil characteristics are means (*n* = 6) ± 1 standard error.

Site climate characteristics were estimated from local weather station data managed by the Western Regional Climate Center (http://www.wrcc.dri.edu). Long-term weather information (1897–2016 mainland; 1909–2016 island) was available from stations within 10 km and at similar elevations to both field sites. In addition, site-level temperature and humidity (at 30-min interval) were recorded during the 3-year study using HOBO data loggers placed at each site (Onset Computer Corporation, Bourne, MA, USA). Similarity of soil characteristics was determined by analyzing soil particle size (% sand, % silt and % clay) and soil fertility (Total N and Total C) in the top 10 cm (Table 1). Soil samples (*n =* 6) were collected within 2 days from both island and mainland field sites and transported to UC Berkeley for processing. Samples were analyzed by the UC Davis Analytical Labs (https://anlab.ucdavis.edu/analysis/Soils/320). In brief, Total *N* and Total *C* were quantitatively determined via a combustion method with a thermal conductivity detector (TCD) system and an IR detector. This method is based on the oxidation of the sample during flash combustion that converts nitrogen and carbon substances into combustion gases and has a detection limit of 0.02% for C and N (AOAC Official Method 972.43, 1997). In addition, % sand, % silt and % clay were determined based on settling rates in an aqueous solution using a hydrometer (Sheldrick and Wang, 1993). The chief difference between sites was the strength of the maritime influence on temperature seasonality, with the island site experiencing more moderate conditions—cooler summers and warmer winters—compared to the more seasonally variable and extreme mainland site (Fig. 2).

Ten phylogenetically independent island–mainland taxon pairs (seven congeneric and three conspecific pairs) were used in this study (Fig. 3). The 10 island–mainland pairs were spread across five plant families and were representative of the dominant lineages in southern California chaparral. Included in these pairs were several species endemic to the Channel Islands (*Prunus ilicifolia* ssp. *lyonii*, *Quercus pacifica*, *Ceanothus megacarpus var. insularis* and *Ceanothus arboreus*) and one species endemic to Santa Catalina Island (*Arctostaphylos catalinae*). With one exception, all island–mainland pairs belonged to distinct genera; in the genus *Ceanothus* taxon pairs were drawn from each of the two distinct subgenera (*Ceanothus-Ceanothus* and *Ceanothus-Cerastes*), which often exhibit different functional and life history traits (McMinn, 1942; Nobs, 1963; Ackerly *et al.,* 2006; Fross and Wilken, 2006; Pratt *et al.,* 2008; Burge *et al.,* 2011). Because the experimental design includes comparisons of both congeneric and conspecific pairs, we will refer to all species pairs as ‘taxa’ or ‘taxon pairs’ for clarity.

### Seasonal water relations and chlorophyll fluorescence

To determine plant water status, monthly to bi-monthly measurements (March 2012–March 2013) of leaf water potential (Ψ_w_) were estimated using a pressure chamber (PMS Instrument Company, Albany, OR, USA). At predawn (4—6 am; Ψ_pd_) and midday (12—2 pm, Ψ_md_), leaves of six individuals per taxon (6 indiv. × 10 taxa = 60 samples at each site) were harvested, bagged and placed in an ice chest. Samples were immediately (typically within 30 min.) used to estimate leaf water potential in the field using pressure chambers and attached portable N tanks. Care was taken to select the youngest healthy, fully mature leaves and branchlets exposed to full sun. In addition to analyzing seasonal patterns in Ψ_pd_ and Ψ_md_, the minimum water potential (Ψ_min_) measured at midday during the end of the dry season (September 2012) was compared between island–mainland taxon pairs. Measurements were always recorded the same day within a site and within 2 days across sites, using the same techniques and equipment.

Stomatal conductance was measured during the study (June 2012–March 2013) using a steady-state leaf porometer (SC-1, Decagon Devices, Pullman, WA). Measurements were performed on six fully mature, sun-exposed leaves per taxon (6 indiv. × 10 taxa = 60 measurements). To account for diurnal fluctuations in atmospheric and solar condition, these measurements were always conducted during the same time of day (9 am—12 pm), during mostly clear (i.e. cloud-free) days. Minimum values of stomatal conductance measured at the end of the dry season (September 2012) were compared across island and mainland sites.

To determine drought stress effects on leaf photosynthetic capacity, intrinsic quantum efficiency of PSII (*F*_v_/*F*_m_) was measured at midday during the peak of the dry season (September 2012) using a pulse-modulated chlorophyll fluorometer (FMS2, Hansatech, Pentney, Norfolk, UK). Measurements were conducted on the same individuals measured for seasonal water relations. Prior to measurements, leaves were dark-adapted for 15–20 min using dark adaptation leaf clips (Hansatech, Pentney, Norfolk, UK). Initial fluorescence (*F*_o_) was measured using low levels of light followed by a saturating pulse of light (15 000 μmols m^−2^ s^−1^) to measure maximum fluorescence (*F*_m_). Variable fluorescence (*F*_v_) was calculated as initial minus maximum fluorescence and intrinsic quantum efficiency of PSII was expressed as *F*_v_/*F*_m_.

### Hydraulic traits and safety margins

To determine the sensitivity of stem xylem to drought conditions, cavitation resistance was estimated with vulnerability curves using a standard centrifuge technique with a conductivity apparatus (Alder *et al.,* 1997; Tobin *et al.,* 2013; Supplementary Fig. S8). Stems approximately 5–6 mm in diameter were harvested from the same six individuals per taxon used for seasonal water relations. The samples were bagged and transported to a laboratory where they were refrigerated until measurements could be performed (within 7 days). Prior to measurements, stems were cut to either 140 or 270 mm long and flushed for 60 min at 100 kPa to remove emboli with an ultra-filtered (0.1 μm pore exclusion filter) solution of deionized and degassed 20 mM KCl solution. The longer stem lengths were used for taxa that had extremely high resistance to cavitation (e.g. *Ceanothus spp*.) and spun in a larger centrifuge rotor capable of delivering greater levels of centrifugal force to the water column. In some cases, stems were rehydrated overnight under a vacuum using the same degassed 20 mM KCl solution, in place of flushing. Following flushing (or vacuum rehydration), hydraulic conductivity (*K*_h_) of stem xylem was measured using a tubing apparatus under a low-pressure head (about 4 kPa). This gave the maximum *K*_h_ (*K*_hmax_) with xylem emboli removed. Stems were then spun in a centrifuge to generate negative xylem tension and repeatedly measured to determine loss of *K*_h_ with decreasing water potential. Percentage loss of *K*_h_ (PLC) was calculated as:

$$ \mathrm{Loss}\kern0.17em \mathrm{of}\;{K}_{\mathrm{h}}\left(\%\right)=\left(1-{K}_{\mathrm{h}}/{K}_{\mathrm{h}\mathrm{max}}\right)\times 100 $$

Vulnerability curves were constructed by plotting decreasing values of water potential versus PLC (supplementary materials). Stem-specific hydraulic conductivity (*K*_s_) and cavitation resistance (P_50_) were estimated from each vulnerability curve. P_50_ was calculated as the water potential at which 50% of hydraulic conductivity (*K*_h_) was lost due to cavitation of xylem conduits. *K*_s_ was calculated by dividing the maximum hydraulic conductivity by the xylem cross-sectional area (mm^2^). Measurements of P_50_ were combined with the minimum seasonal water potential measured at the end of the 2012 dry season (Ψ_min_) to calculate the hydraulic safety margin (HSM_50_) for each taxon (*n =* 6):

$$ {\mathrm{HSM}}_{50}={\Psi}_{\mathrm{min}}-{\mathrm{P}}_{50} $$

Vulnerability curves were measured on 9 of the 10 island–mainland taxon pairs in two sampling efforts. Five pairs were measured in Summer/Fall 2010 (*Arctostaphylos, Ceanothus-Ceanothus, Ceanothus-Cerastes, Heteromeles* and *Quercus*) and four pairs were measured in Summer/Fall 2012 (*Adenostoma, Cercocarpus, Prunus, Rhus*). Because vulnerability curves were not calculated for *Rhamnus ilicifolia*, due to a sampling error, the *Rhamnus* pair was removed from this analysis. While measurements of vulnerability curves were performed in different years, measurements within taxon pairs were always performed in the same season and year. Therefore, seasonal and inter-annual variation in cavitation resistance (Jacobsen *et al.,* 2007a,b, 2014) should not affect comparisons within taxon pairs.

Some of the taxa measured have known vessel lengths longer than the excised stems used here to measure vulnerability curves (Jacobsen *et al.,* 2012). Recent work has demonstrated that the ‘long vessel artefact’ (Cochard *et al.,* 2010) is not an issue when using standard centrifuge methods like those presented here (Tobin *et al.,* 2013; Hacke *et al.,* 2015; Pratt *et al.,* 2019; Jacobsen *et al*. 2019; Jacobsen and Pratt *et al.,* 2012; Sperry *et al.,* 2012). However, some readers may still be concerned about such an effect (Skelton *et al*., 2018). Therefore, in order to demonstrate our findings are robust in the face of current debates on methodology, we have analyzed our hydraulic trait data (P_50_ and HSM_50_) in multiple ways to illustrate the effects of different curve shapes on our main findings, which are consistent across alternative analytical approaches (see Supplementary Table S3).

Xylem density (XD) was measured by dividing the dry mass of xylem tissue by its water-saturated volume. To measure XD, ~5 cm long segments were cut from the same stems used to construct vulnerability curves. The segments were cut longitudinally and the pith and bark were removed manually. The segments were then soaked overnight in degassed water brought to a pH of 2. The volume of fully saturated stem segments was determined using Archimedes principle. Following volume measurements, stem segments were dried to a constant weight in a drying oven and dry mass was determined using a four-digit balance.

### Remote sensing of drought response

In order to determine if observed trait patterns were associated with broader vegetation responses to recent drought events in our study area, we analyzed the Enhanced Vegetation Index (EVI) derived from Landsat surface reflectance data (NASA/USGS; 30 m resolution), accessed via the Climate Engine web tool (Huntington *et al.,* 2017). EVI is similar to the Normalized Difference Vegetation Index (NDVI), widely used as an index to monitor vegetation health during drought (e.g. Byer and Jin 2017), but optimizes the vegetation signal by reducing noise from canopy background and atmospheric conditions (Liu and Huete, 1995). We generated time series of mean summer EVI (June–August) from 2000 to 2017 for the three California Channel Islands with significant chaparral components (Santa Catalina, Santa Cruz, and Santa Rosa Island). We also generated summer EVI values for chaparral-dominated areas in three south coast mountain ranges (Santa Monica, Santa Ynez, and Santa Ana mountain ranges). These areas were selected because they occur at similar latitudes, elevational ranges, and have similar vegetation composition as the three California Channel Islands analyzed (Supplementary Fig. S1).

We categorized the EVI data into drought and non-drought years based on the U.S. Drought Monitor categorizations for the south coast region. Non-drought years were used to calculate an EVI ``baseline'' for each area. This baseline was then used to quantify departures of EVI from normal conditions by calculating *z*-scores:

$$ z=\frac{{\mathrm{EVI}}_{\mathrm{year}}-{\mathrm{EVI}}_{\mathrm{baseline}}}{\sigma_{\mathrm{baseline}.}} $$

where EVI_baseline_ and σ_baseline_ represent the mean and interannual standard deviation for baseline years and EVI_year_ is the mean summer EVI value for a given year. This *z*-score-based method has recently been shown to track drought-induced declines in vegetation health in the Sierra Nevada Mountains, California, during the recent multi-year drought (Byer and Jin 2017).

Mean z-scores for island and mainland areas were computed and compared for two drought periods: the single-year drought of 2007 and the recent multi-year drought (2012–2017). Special attention was paid to the EVI patterns during and immediately following these two drought events to compare both the drought resistance (magnitude of decline in EVI during drought) and resilience (magnitude of EVI improvement post-drought) of island and mainland vegetation.

### Statistical analyses

Seasonal water potential and stomatal conductance were analyzed using repeated-measures ANOVA with site (island/mainland), taxon-pair and date as independent variables and plant ID (i.e. individual) as a random, nested variable (Supplementary Table S1). Mean values for minimum stomatal conductance, minimum water potential, chlorophyll fluorescence, cavitation resistance, xylem density and hydraulic safety margins were compared using a mixed-model ANOVA with site (island/mainland) as a fixed factor and taxon-pair as a random variable nested within site. This model was used to test for general differences between island–mainland pairs (Supplementary Table S2). Additional pairwise comparisons were used to test for differences within species pairs when a significant site × taxon interaction was detected. A two-way ANOVA was used to compare mean EVI *z*-scores between island and mainland environments, and pairwise comparisons were used to test for differences during specific drought and post-drought years.

## Results

### Seasonal water relations

**Figure 4:**
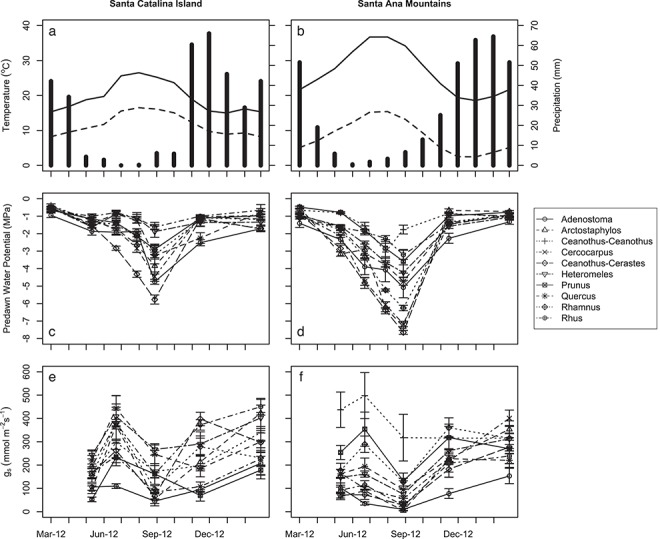
Figures showing contrasting climate (**a**, **b**), seasonal water potential (**c**, **d**) and stomatal conductance (**e**, **f**) for island (a, c, e) and mainland (b, d, f) field sites. Climate figures depict monthly averages of max. Temperature (solid line), min. Temperature (dashed line) and precipitation (vertical bars) based on long-term climate data from nearby weather stations (1908–2019). Water potential and stomatal conductance plots depict mean values ±1 SE for each species.

Predawn water potential (Ψ_pd_) varied throughout the study (Fig. 4, Supplementary Table S2, Supplementary Figs S4 and S5) with maximum (least negative) values (Ψ_max_) recorded during the wet season (March 2012 and 2013) and minimum (most negative) values (Ψ_min_) recorded during the peak of the dry season (September 2012). Within each site, taxa differed in Ψ_pd_, especially during the dry season (Fig. 4, Supplementary Figs S4 and S5). Shallower-rooted, more cavitation resistant taxa (e.g. *Ceanothus-Cerastes* and *Arctostaphylos*) exhibited more negative Ψ_pd_ than deeper-rooted, less cavitation resistant taxa (e.g. *Heteromeles*, *Quercus*, and *Rhus*). Differences across sites were 2 MPa lower for mainland vegetation on average (Supplementary Fig. S5), but varied by taxon pair, resulting in a significant site × taxon interaction (Supplementary Table S1). Stomatal conductance (*g*_s_) exhibited little variation seasonally (Fig. 4, Supplementary Table S1). However, for most taxa, the lowest values of *g*_s_ were measured during the peak of the dry season (Fig. 4), suggesting plants experienced drought-induced stomatal closure. Differences across sites varied by taxon pair, resulting in a significant site × taxon interaction (Supplementary Table S1).

### Minimum seasonal water potential, stomatal conductance and chlorophyll fluorescence

**Figure 5:**
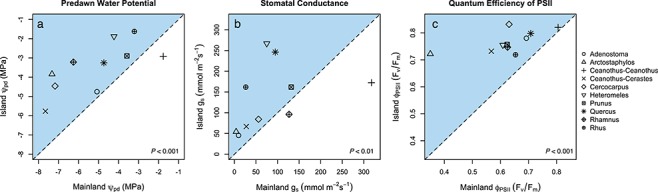
Predawn water potential (**a**), stomatal conductance (**b**) and quantum efficiency of PSII (**c**) measured during the peak of the summer dry season for 10 island–mainland species pairs. Each point represents one species pair. Dashed line is a 1:1 line. A majority of points above the 1:1 line (i.e. blue region) indicate more favorable water relations for island plants. *P-*values for paired statistical comparisons are depicted in the lower right corner of each plot.

**Figure 6:**
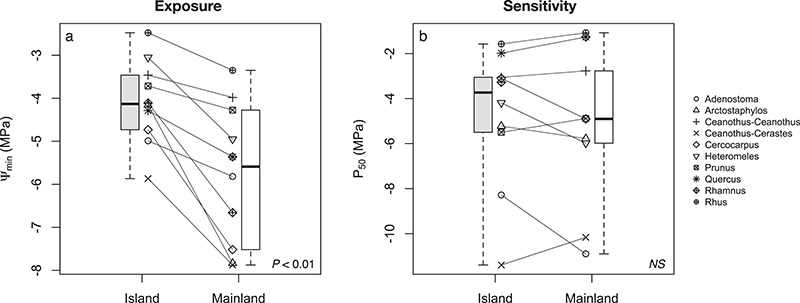
Minimum seasonal water potential (Ψ_min_; **a**) and cavitation resistance (P_50_; **b**) for island–mainland species pairs. These traits serve as proxies for exposure versus sensitivity to drought and are the components used to estimate hydraulic safety margins and provide a quantitative test of the conceptual model linking physiological trait patterns to detection of climate refugia (Fig. 1). *P-*values for paired statistical comparisons are depicted in the upper left corner of each plot.

During the peak of the dry season (September 2012), predawn water potential (Ψ_pd_), stomatal conductance (*g*_s_) and chlorophyll fluorescence (*F*_v_/*F*_m_) were higher (more favorable) for island plants compared to mainland relatives (Fig. 5, Supplementary Table S1; *P* < 0.001). Generally, deep-rooted taxa that resprout after fire (e.g. *Rhus, Heteromeles, Prunus*) had the highest (least negative) Ψ, while more shallow-rooted, obligate seeders (e.g. *Ceanothus-Cerastes*, *Arctostaphylos*) had the most negative water potentials (Figs 5a, 6a, Supplementary Table S2, Supplementary Figs S4 and S5). Deep-rooted resprouters also had the largest differences in g_s_ across sites (Fig. 5b, Supplementary Table S2), but the largest differences in *F*_v_/*F*_m_ were observed in non-sprouting *Arctostaphylos* (Fig. 5c, Supplementary Table S2).

### Hydraulic traits and safety margins

None of the measured stem hydraulic traits (K_s_, P_50_, or XD) were consistently different between island—mainland sites (*P* > 0.05; Supplementary Table S2). Significant pairwise comparisons of *K*_s_ were recorded for *Ceanothus-Ceanothus*, *Adenostoma*, *Cercocarpus* and *Prunus*. Resistance to drought-induced cavitation (P_50_) varied widely between taxa, with similar ranges at each site (−1 to −11 MPa; Fig. 6). However, there were no consistent differences between island–mainland taxon pairs (*F*_1,94_ = 3.952; *P* = 0.117). Only *Heteromeles* exhibited a significant pairwise comparison, with greater resistance on the mainland (*P* < 0.05; Supplementary Table S2). Xylem density, XD, was also not consistently different between island–mainland relatives (*F*_1,109_ = 0.405; *P* = 0.526). None of the pairwise comparisons of XD were significantly different (*P* < 0.05; Supplementary Table S2).

**Figure 7:**
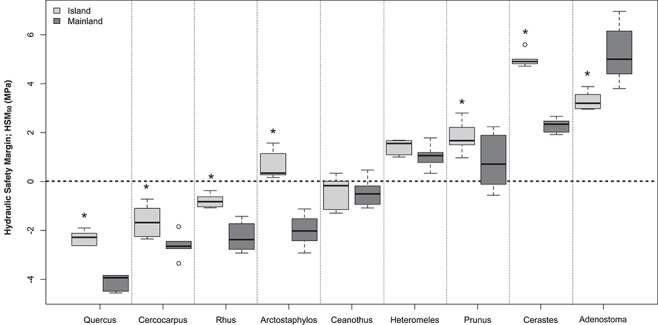
Box plots of hydraulic safety margins (HSM_50_ = Ψ_min_—P_50_) for nine island-mainland species pairs. Blue background indicates higher safety margin for island species (8/9 pairs); red background indicates lower safety margin for island species (1/9 pairs). Asterisks denote significant pairwise comparisons (*P* < 0.05).

Hydraulic safety margins (HSM_50_) also varied widely between taxa (−4 to +5 MPa; Fig. 7). Nine of 18 taxa measured had negative safety margins, suggesting they are likely to experience > 50% loss of hydraulic conductivity during the peak of the dry season (some of the negative safety margins may reflect long-vessel artifacts affecting the P50 values). In general, taxa maintained the same ranking in safety margin across sites—i.e. taxa with high safety margins relative to other taxa on the mainland also had high safety margins on the island. Importantly, island taxa had consistently higher safety margins (*F*_1,94_ = 37.950; *P* < 0.01) with significant pairwise comparisons in 7 out of 9 taxon pairs (Fig. 7). Our findings of no difference in P_50_ and strong differences in HSM_50_ between island–mainland taxa are consistent whether or not long-vesseled taxa are included in the analysis (Table S3).

### Remote sensing of responses to recent drought

**Figure 8:**
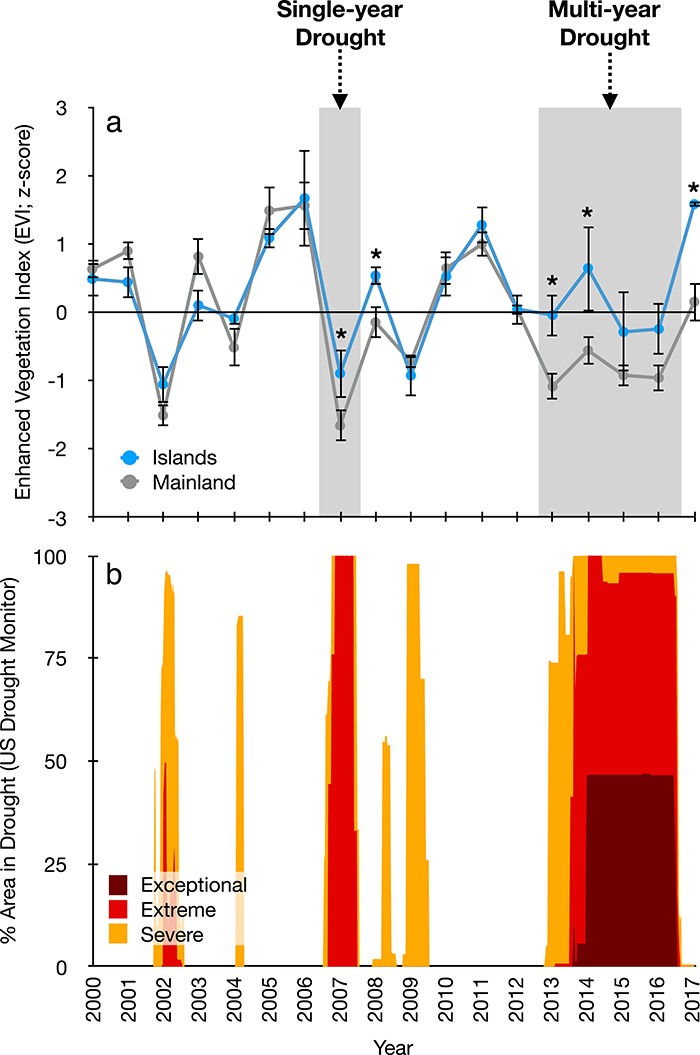
Time-series of mean summer (JJA). Enhanced Vegetation Index in southern California island and mainland environments (**a**). Recent acute (single year; 2007) and chronic (multi-year; 2012–2017) drought events are shaded in gray. Lower panel (**b**) shows U.S. Drought Monitor categorizations for the South Coast region (*U.S. Drought Monitor:*http://droughtmonitor.unl.edu/) during the same time interval. Island EVI is averaged from Santa Catalina, Santa Cruz and Santa Rosa Islands, which all have significant island chaparral components. Mainland EVI is averaged from chaparral-dominated areas in the Santa Monica, Santa Ynez and Santa Ana ranges. EVI averages were generated by combining surface reflectance data from Landsat 4/5/7/8 using the Climate Engine web tool (https://app.climateengine.org/). Asterisks denote significant differences during drought years and years immediately following drought (i.e. recovery years).

Interannual variation in mean summer EVI *z*-scores allowed for meaningful comparison of island and mainland vegetation responses to recent drought events (Fig. 8). During the extreme drought conditions across southern California that took place in 2007 (single-year drought), island plants experienced less-pronounced reductions in summer EVI compared to mainland areas (*P* < 0.05). In addition, the island vegetation rebounded more quickly in the first year after the drought (*P* < 0.05). The patterns were similar during the recent ‘exceptional’ multi-year drought (2012–2016). However, during the third and fourth years of the drought, increased variability in island EVI resulted in no statistically significant differences between island and mainland environments during these years. The island plants also had more favorable EVI response following the multi-year drought (*P* < 0.05).

## Discussion

### Conservative traits result in greater hydraulic safety for island plants

We did not observe consistent differences in cavitation resistance (P_50_) between island–mainland sites. This lack of a difference in P_50_ between sites with different levels of water availability is surprising considering global meta-analyses that show P_50_ values closely match environmental patterns of water availability (e.g. Choat *et al.,* 2012). This ‘fine-tuning’ of plant hydraulics to current environmental conditions suggests that on a global scale plants are similarly at risk of hydraulic failure, despite differences in water availability and functional strategies. However, our findings suggest that there may be important exceptions on a regional to local scale, where P_50_ does not closely track patterns of water availability. This is consistent with studies comparing hydraulic traits between intraspecific populations in coastal and drier interior sites (Jacobsen and Pratt, 2013; Jacobsen *et al.,* 2014), as well as community-level analyses of different semi-arid plant communities (Jacobsen *et al*., 2007a).

It is not clear why island taxa possess levels of cavitation resistance similar to mainland relatives living in drier environments. One hypothesis for the disparity between water availability and P_50_ is that cavitation resistance arises from selection at the seedling stage, when the risk of drought-induced cavitation is high due to a small, developing root system’s need to provide enough water for a rapidly growing shoot (Frazer and Davis, 1988; Thomas and Davis 1989; Schwilk and Ackerly, 2005; Pratt *et al.,* 2008). Another hypothesis is that cavitation thresholds are set during severe episodic droughts (Pockman and Sperry, 2000), suggesting that water availability and P_50_ may be decoupled during more normal conditions. However, in order for these hypotheses to explain the patterns in the present study, water stress would have to be similar between island and mainland plants at the seedling stage but not at the adult stage, or island and mainland plants would need to experience similar conditions during episodic droughts but not during the intervening years. Neither of these explanations seems very likely considering the consistent gradient in climate that exists between the island and mainland environments (Fig. 2; Weigelt *et al.,* 2013).

Another factor that may explain the lack of P_50_ differences between island–mainland pairs is the weak relationship between hydraulic safety and hydraulic efficiency. In studies that account for phylogenetic similarity, no correlation between P_50_ and hydraulic conductivity (*K*_s_) is typically observed (Maherali *et al.,* 2004; Bhaskar *et al.,* 2007; Jacobsen *et al.,* 2007b). Therefore, it is possible that the trade-offs between hydraulic safety and efficiency are not strong enough to drive selection against high cavitation resistance once it has evolved in a lineage. Consistent with this are studies that have shown cavitation resistance to be a highly conserved trait (Lamy *et al.,* 2011; Hao *et al.,* 2008; Wilson *et al.,* 2008; Pittermann *et al.,* 2012). In the present study, we are comparing island lineages, most likely descended from mainland taxa that migrated to the islands to their contemporary mainland relatives. Colonization of the island by mainland taxa occurred at some point during the approximately 500 000 years that the island has been continuously above water (Schoenherr *et al.,* 1999). It is possible that island plants have retained the high cavitation resistance of their mainland ancestors due to weak selection against it, coupled with the relatively short time that they have been isolated on the island environment.

In general, few studies examine the spatial and temporal variation in hydraulic traits (Anderegg, 2015). Of those that do, similar evidence of low variability across environmental gradients is often observed (e.g. Jacobsen and Pratt, 2013; Jacobsen *et al.,* 2014; Skelton *et al*., 2019). The observed low spatial variability in hydraulic traits may be explained, in part, by high phylogenetic conservatism in such traits (Lamy *et al.,* 2011; Hao *et al.,* 2008; Pittermann *et al.,* 2012; Skelton *et al*., 2018), This suggests that many plants may be relatively protected from hydraulic failure compared to close relatives or ecotypes in comparatively drier environments. Therefore, hydrologic refugia capable of buffering plants from hydraulic failure may be common in nature.

The lack of variation in hydraulic traits does not preclude variation in other important functional traits that allow island taxa to achieve a ‘fit’ with their contemporary environment. Hochberg (1980) first analyzed differences in leaf traits between island and mainland shrubs native to southern California, finding that island plants exhibited more mesomorphic (i.e. drought-sensitive) traits. In addition, Bowen and Vuren (1997) found that plants from Santa Cruz Island, California, possessed less robust leaves that made them preferred forage for exotic herbivores. Salladay and Ramirez (2018) found similar results for leaves of several island plant species on Santa Catalina Island. Either stronger selection or greater phenotypic plasticity in leaf traits may explain the differences observed between these previous studies and the current one.

### Island plants are buffered from seasonal and interannual drought conditions

Despite similar precipitation regimes, island plants had higher water availability and experienced less water stress during the dry season as evidenced by higher (less negative) water potentials, higher stomatal conductance and higher chlorophyll fluorescence. Furthermore, remotely sensed health (via EVI analysis) of island plants was less impacted by recent drought events. These findings are consistent with a recent study comparing the dry season water relations of *Arctostaphylos* spp. in maritime versus interior chaparral sites in California (Vasey *et al.,* 2012; Jacobsen and Pratt, 2013). In that study, water relations differences between maritime and interior *Arctostaphylos* populations were explained by the reduced evaporative demand and increased inputs from fog that are characteristic of coastal California environments. It is likely that these factors—reduced evaporative demand and increased summertime fog—also affect plant water relations on the California Channel Islands (Williams *et al*., 2008b; Fischer *et al.,* 2009; Carbone *et al.,* 2011; Taylor *et al.,* 2019; Supplementary Fig. S3).

Another factor that may be related to dry season water relations in island and mainland sites is reduced shrub density resulting from prior land use patterns. Island chaparral communities on SCI are less dense, exhibiting a more open canopy structure than mainland chaparral communities (Hochberg, 1980; Minnich, 1982; Schoenherr *et al.,* 1999; Supplementary Fig. S6). This pattern is thought to largely be an artifact of 19th and 20th century land use practices on SCI—specifically, overgrazing by feral animals—and may not reflect the ‘natural’ state of SCI plant communities (Minnich, 1982; Rick *et al.,* 2014). Previous studies in southern California chaparral and coastal sage communities have shown that decreased transpiration and improved water availability are associated with low woody vegetation density (Ng and Miller, 1980; Poole and Miller, 1975).

Reducing stand density and competition for water is hypothesized to improve individual plant water relations and is a theoretical justification for the thinning of forest stands as a strategy for managing drought impacts (Grant *et al.,* 2013; McDowell and Allen, 2015; Bradford and Bell, 2017). It is possible that a similar process takes place in SCI chaparral communities where the altered canopy structure (i.e. reduced stand density) results in reduced competition by woody plants for deeper water sources, leaving more water available to the remaining individuals in the community (Hochberg, 1980). However, recent work from another semi-arid system finds little evidence for this ‘moisture release hypothesis’, suggesting instead that any reductions in competition are offset by increased water loss from exposed soil, wind and changes in hydraulic redistribution (Morillas *et al.,* 2017). It should also be noted that the potential role of introduced herbivores in improving outcomes of island plants is limited. Ramirez *et al.* (2012) found that browsing by mule deer on Santa Catalina Island caused high post-fire mortality—presumably, via carbon starvation—despite these populations having a reduced risk of hydraulic failure compared to the mainland. Future studies designed to isolate the effects of maritime climate and stand density may help determine their independent roles in determining seasonal water relations of plants in this system.

While increased buffering of mature island chaparral shrubs was observed in the present study, drought stress may still play an important role in island sites through impacts to post-fire seedlings and resprouts, as well as localized impacts on drought-sensitive species. In a recent study of three island chaparral species from Catalina Island, Jacobsen *et al*. (2018) found that seedlings recruiting after a recent fire experienced high levels of mortality and that cavitation resistance was predictive of these mortality patterns. In addition, indirect effects of drought such as increased browsing pressure on delicate regenerating vegetation during drought years may contribute to post-fire mortality patterns (Ramirez *et al.,* 2012). On Santa Cruz Island, southern California, bishop pines (*Pinus muricata* D. Don) have experienced drought-related mortality during recent drought events (Baguskas *et al.,* 2016; Taylor *et al.,* 2019). Furthermore, there may be limits to the buffering observed in the present study for drought-tolerant chaparral species that will be crossed during continued warming, drying climate trends. Like other climate change refugia, the persistence of species and ecosystems in hydrologic refugia may only provide a temporary climate buffer (Millar *et al.,* 2007; Morelli *et al.,* 2016; McLaughlin *et al.,* 2017).

### Hydraulic safety reveals hydrologic refugia

The improved safety margins we observed in island chaparral shrubs may allow them to fare better during episodes of increasing aridity. Greater hydraulic safety in island plants indicates that they are able to tolerate greater declines in minimum seasonal water potential before they experience the same amount of drought-induced cavitation as mainland relatives. Our remote sensing of island vegetation responses during recent droughts suggests this buffering may improve drought resistance and resilience in these environments. Therefore, as the regional patterns of climate change in California trend towards warmer, drier conditions, chaparral shrubs living on the California Channel Islands may experience less drought-induced cavitation, fewer declines in performance and lower rates of mortality compared to mainland plants with lower hydraulic safety margins.

Improved safety margins may also have been a factor during past episodes of climate change, contributing to the pattern of relictual endemism on the California Channel Islands. Many of the woody endemics on the California Channel Islands are thought to be remnant populations of lineages that once had broader distributions (Axelrod, 1967; Raven and Axelrod, 1978; Schoenherr *et al.,* 1999). The general explanation offered for this pattern of relictual endemism on the California Channel Islands is that changing climatic conditions since the late Tertiary has resulted in extirpation of mainland populations and persistence of island populations due to more favorable climate and reduced competition in insular environments. Our findings allow for such explanations to be taken a step further by suggesting that reduced risk of hydraulic failure may have contributed to woody plant lineages persisting on the California Channel Islands during past transitions to warmer, drier climates.

In conclusion, insular plant communities off the coast of southern California have more favorable hydraulic traits that underlie greater resistance and resilience to seasonal and interannual droughts. This buffering is associated with the finding that island plants do not appear to have ‘fine-tuned’ their stem hydraulic traits in response to the higher water availability in their current insular environment and, therefore, have a reduced risk of hydraulic failure. This pattern of improved hydraulic safety in island plants may have contributed to current biogeographic patterns of endemic island lineages and may continue to buffer island plants from hotter, drier conditions associated with anthropogenic climate change in this or other systems. Furthermore, the identification of high safety margin taxa or ecotypes using the comparative methods described here may be a useful approach to guide management efforts aimed at the detection of climate change refugia (Keppel *et al.,* 2011; Morelli *et al.,* 2016). The present study adds to the growing body of literature suggesting that hydraulic traits and hydraulic safety margins, in particular, are a valuable set of tools for predicting the impacts of drought on plant communities and that similar comparisons of hydraulic traits in other ecosystems may reveal additional hydrologic refugia important to species persistence in the face of a warmer, drier future.

## Supplementary Material

revised_coz115
