# Supplementary material for: Plant hydraulic traits reveal islands as refugia from worsening drought
Source: Conserv Physiol. 2020 Jan 29;8(1):coz115. doi: 10.1093/conphys/coz115 (PMC6988607; doi:10.1093/conphys/coz115)
Supplement: revised_coz115 [file revised_coz115.pdf]

Figure 1.

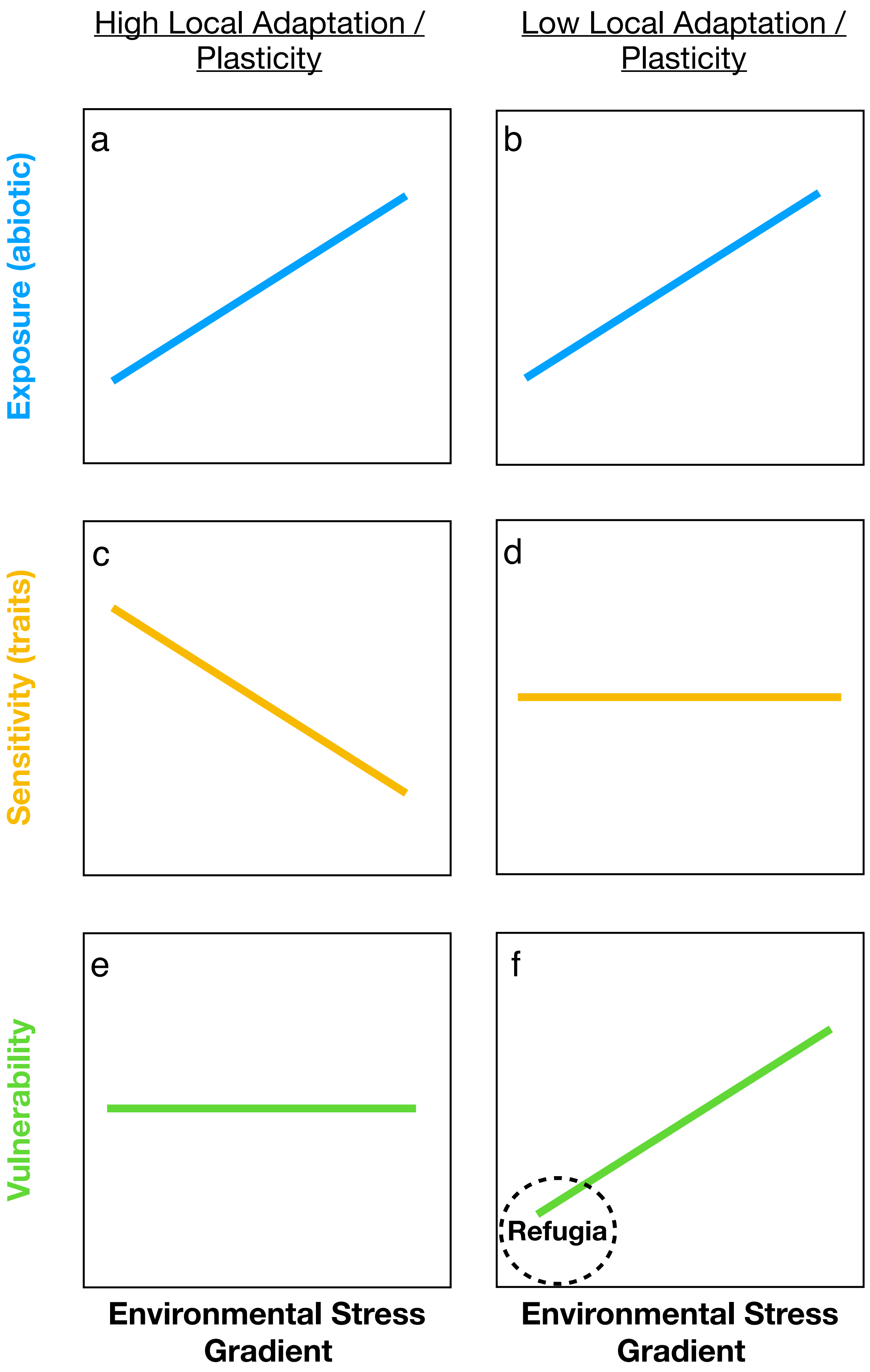

High Local Adaptation / Plasticity
Low Local Adaptation / Plasticity
Exposure (abiotic)
Sensitivity (traits)
Vulnerability
a
b
c
d
e
f
Refugia
Environmental Stress Gradient
Environmental Stress Gradient

Figure 2.

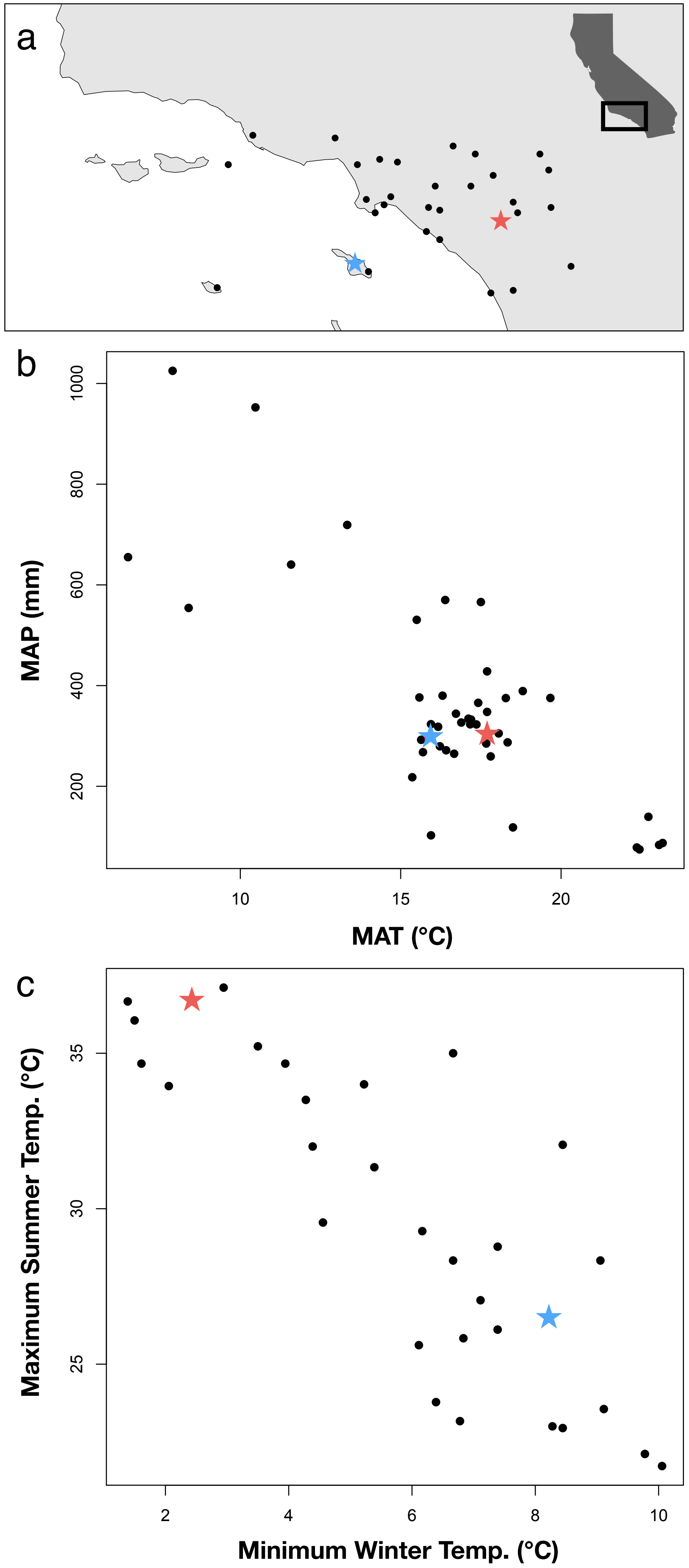
a
b
MAP (mm)
200
400
600
800
1000
10
15
20
MAT (°C)
c
Maximum Summer Temp. (°C)
25
30
35
2
4
6
8
10
Minimum Winter Temp. (°C)

Figure 3.

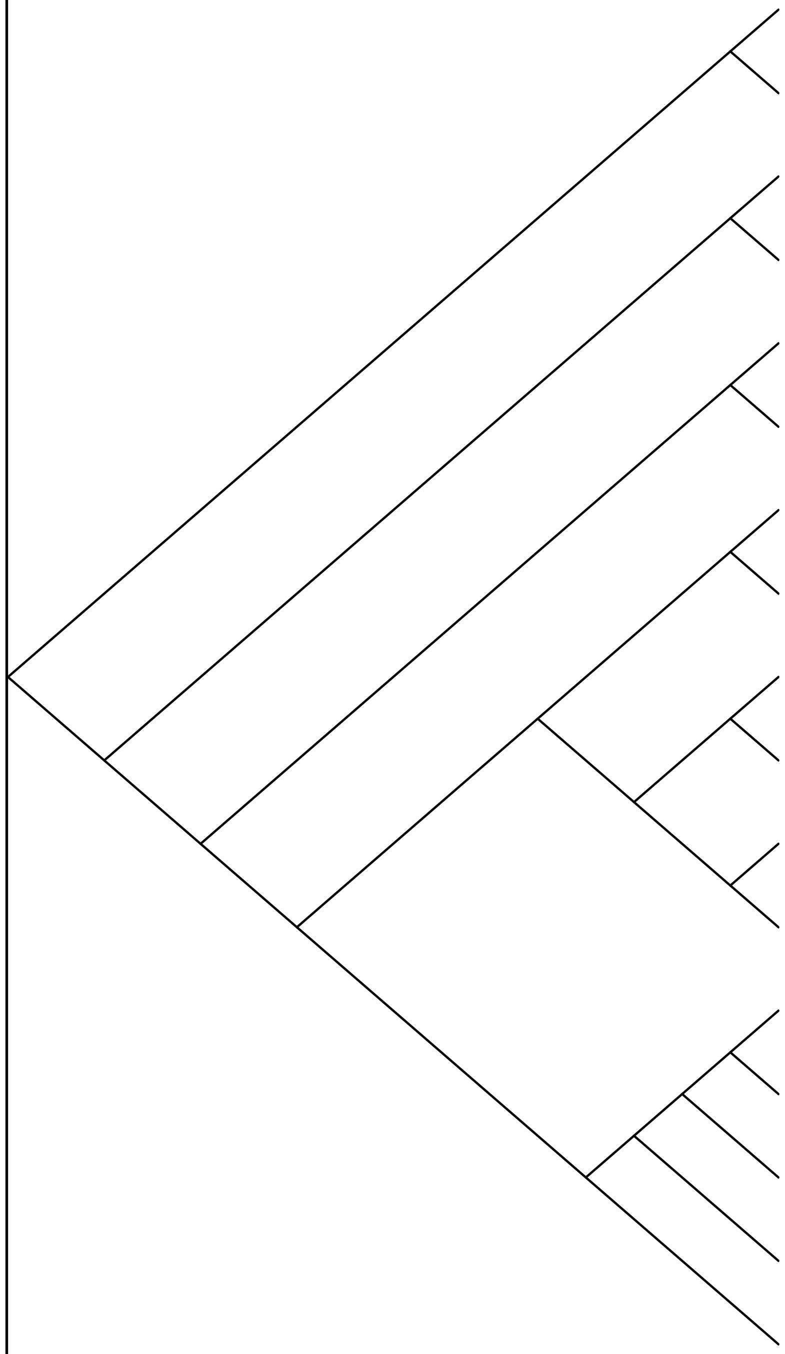

Species	Family	Site
Arctostaphylos glauca Lindl.	Ericaceae	SAM
Arctostaphylos catalinae P.V. Wells**	Ericaceae	SCI
Rhus ovata S. Watson	Anacardiaceae	SAM
Rhus integrifolia (Nutt.) Benth. & Hook. f. ex Rothr.	Anacardiaceae	SCI
Quercus berberidifolia Liebm.	Fagaceae	SAM
Quercus pacifica Nixon & C. H. Mull.*	Fagaceae	SCI
Rhamnus ilicifolia Kellogg	Rhamnaceae	SAM
Rhamnus pirifolia Greene*	Rhamnaceae	SCI
Ceanothus crassifolius Torr.	Rhamnaceae	SAM
Ceanothus megacarpus Nutt. *var. insularis* (Eastw.) Munz*	Rhamnaceae	SCI
Ceanothus oliganthus Nutt.	Rhamnaceae	SAM
Ceanothus arboreus Greene*	Rhamnaceae	SCI
Prunus ilicifolia (Nutt. ex Hook. & Arn.) D. Dietr. *ssp. ilicifolia*	Rosaceae	SAM
Prunus ilicifolia ssp. lyonii (Eastw.) P. H. Raven*	Rosaceae	SCI
Heteromeles arbutifolia (Lindl.) M. Roem.	Rosaceae	Both
Cercocarpus betuloides Nutt.	Rosaceae	Both
Adenostoma fasciculatum Hook. & Arn.	Rosaceae	Both

Figure 4.

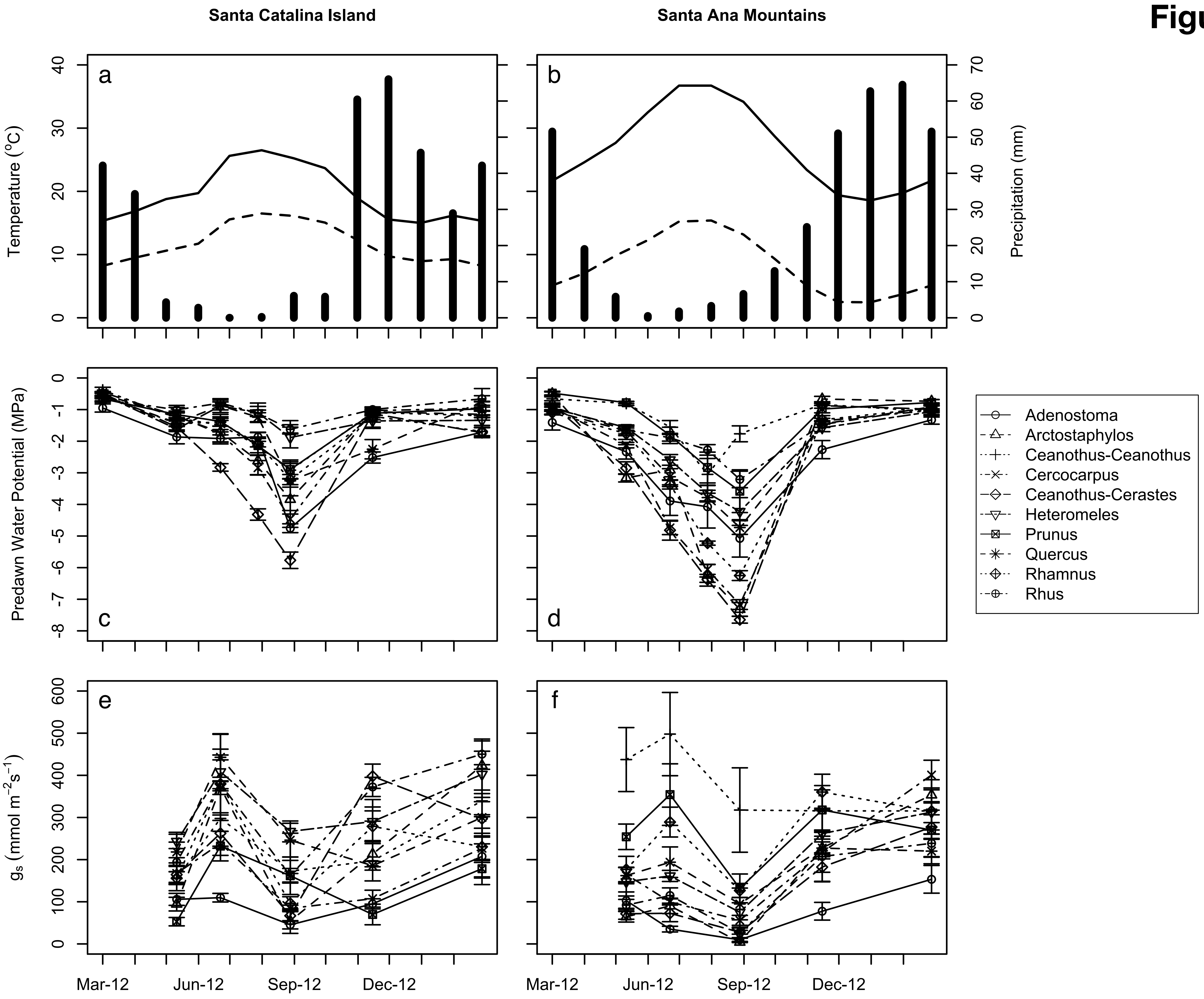
Santa Catalina Island
Santa Ana Mountains
a
b
c
d
e
f
Temperature (°C)
Precipitation (mm)
Predawn Water Potential (MPa)
gs (mmol m−2s−1)
Mar-12
Jun-12
Sep-12
Dec-12
Adenostoma
Arctostaphylos
Ceanothus-Ceanothus
Cercocarpus
Ceanothus-Cerastes
Heteromeles
Prunus
Quercus
Rhamnus
Rhus

Figure 5.

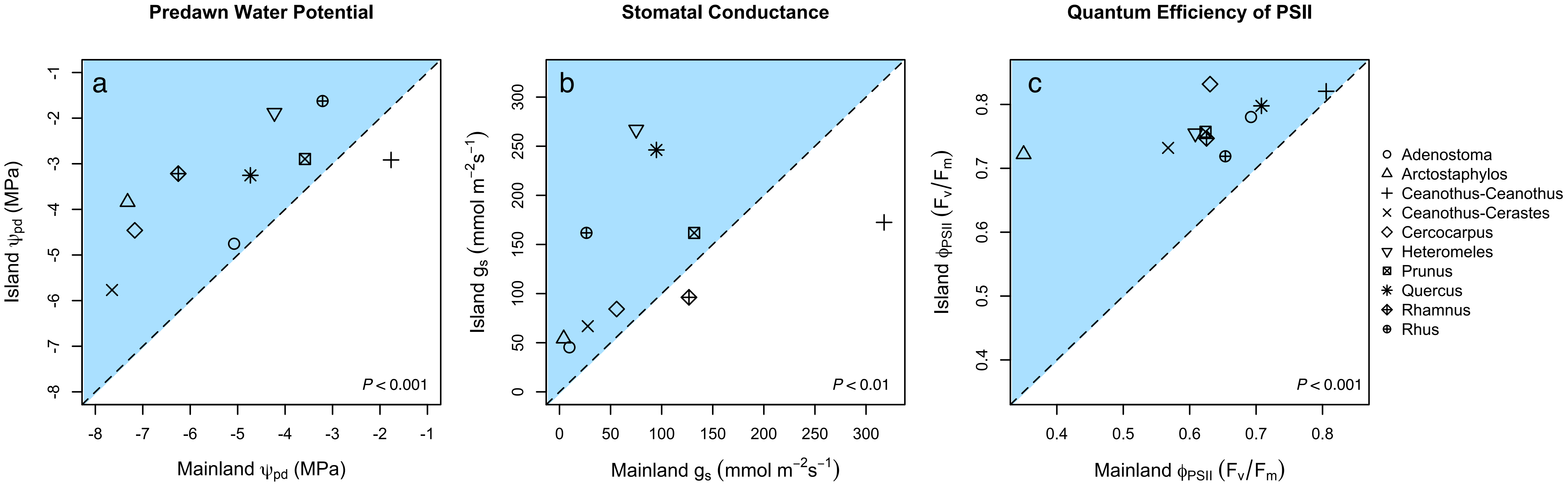

Predawn Water Potential
Stomatal Conductance
Quantum Efficiency of PSII
a
b
c
P < 0.001
P < 0.01
P < 0.001
Island ψpd (MPa)
Mainland ψpd (MPa)
Island gs (mmol m−2s−1)
Mainland gs (mmol m−2s−1)
Island ϕPSII (Fv/Fm)
Mainland ϕPSII (Fv/Fm)
Adenostoma
Arctostaphylos
Ceanothus-Ceanothus
Ceanothus-Cerastes
Cercocarpus
Heteromeles
Prunus
Quercus
Rhamnus
Rhus

Figure 6.

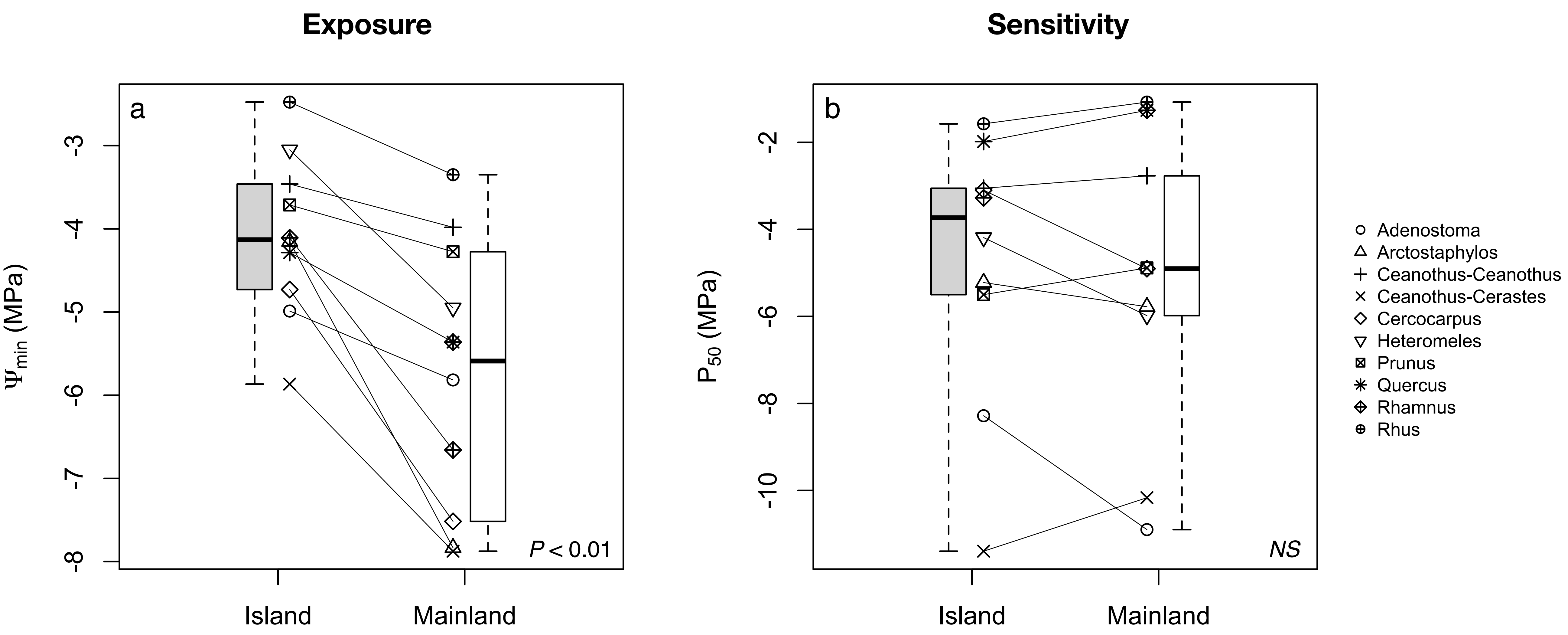

Exposure
Sensitivity
a
b
Ψmin (MPa)
P50 (MPa)
Island
Mainland
P < 0.01
NS
Adenostoma
Arctostaphylos
Ceanothus-Ceanothus
Ceanothus-Cerastes
Cercocarpus
Heteromeles
Prunus
Quercus
Rhamnus
Rhus

Figure 7.

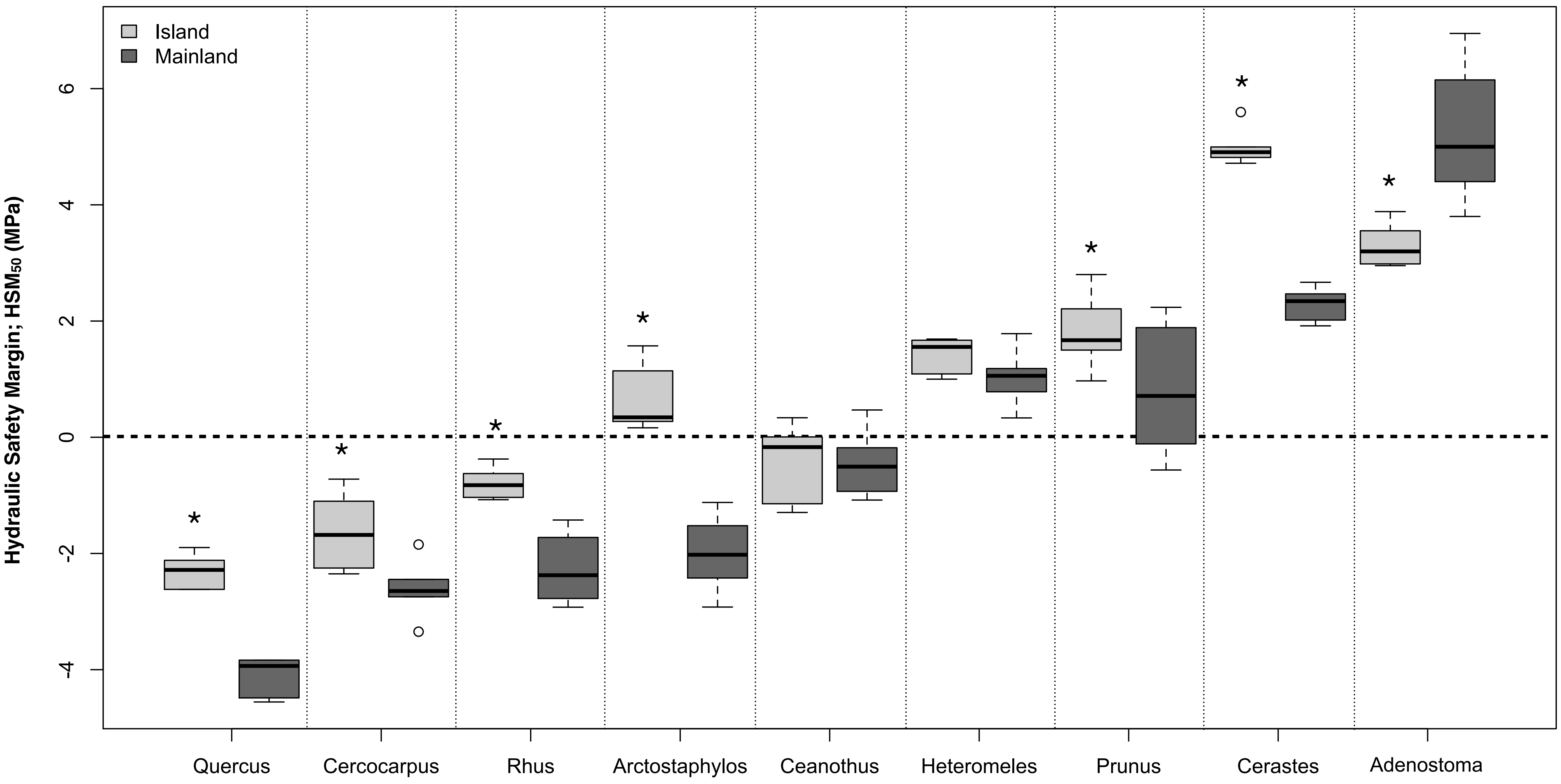
Island
Mainland
Hydraulic Safety Margin; HSM50 (MPa)
6
4
2
0
-2
-4
*
Quercus
Cercocarpus
Rhus
Arctostaphylos
Ceanothus
Heteromeles
Prunus
Cerastes
Adenostoma

Figure 8.

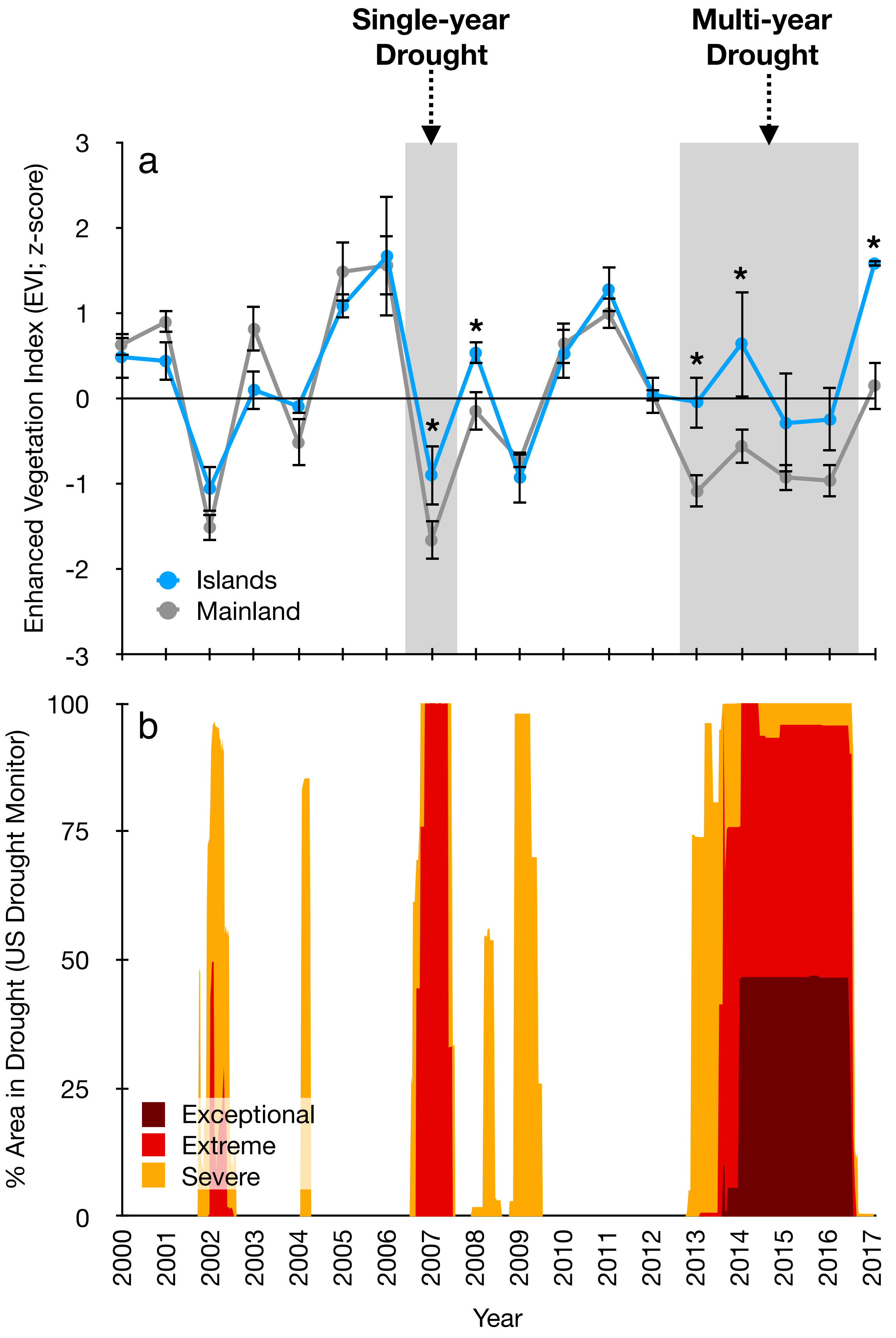

Single-year
Drought
Multi-year
Drought
a
Enhanced Vegetation Index (EVI; z-score)
3
2
1
0
-1
-2
-3
Islands
Mainland
*
b
% Area in Drought (US Drought Monitor)
100
75
50
25
0
Exceptional
Extreme
Severe
2000
2001
2002
2003
2004
2005
2006
2007
2008
2009
2010
2011
2012
2013
2014
2015
2016
2017
Year

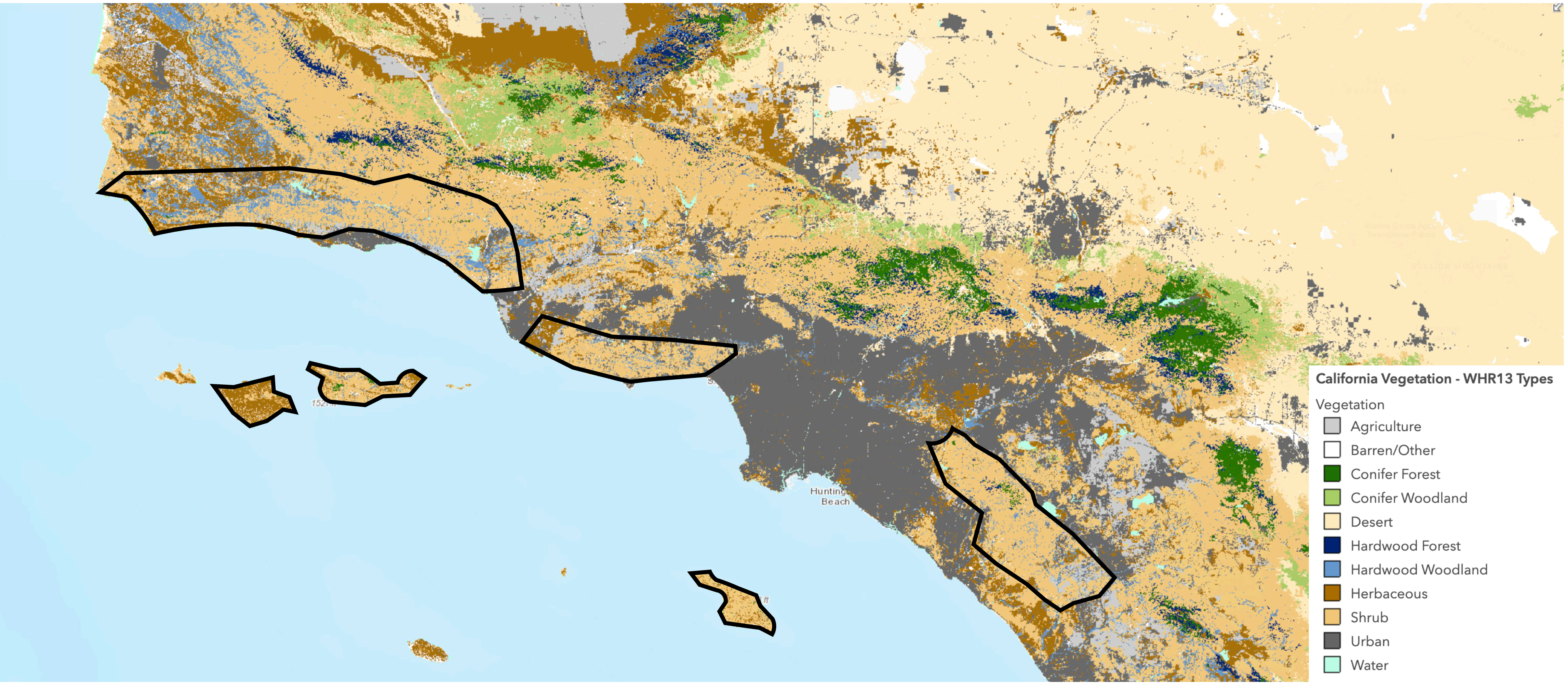

Figure S1**.** Polygons of shrub-dominated (i.e. chaparral) areas used to analyze EVI responses to recent drought events. The three islands (Santa Catalina, Santa Cruz, and Santa Rosa Islands) have the most intact island chaparral components (Schoenherr et al. 1999). The three mainland areas (Santa Monica, Santa Ynez, and Santa Ana mountain ranges) were selected because they occur at similar latitudes and elevational ranges as the three Channel Islands and have similar vegetation composition. Vegetation layer used in selecting areas for analysis is provided by the California Department of Forestry and Fire Protection and represents "*An accurate depiction of the spatial distribution of habitat types within California… The California Department of Forestry and Fire Protection's CALFIRE Fire and Resource Assessment Program (FRAP), in cooperation with California Department of Fish and Wildlife VegCamp program and extensive use of USDA Forest Service Region 5 Remote Sensing Laboratory (RSL) data, has compiled the "best available" land cover data available for California into a single comprehensive statewide data set.*" (accessed via arcgis.online). EVI averages were generated by combining surface reflectance data from Landsat 4/5/7/8 and summarized for each of these areas using the Climate Engine web tool (https://app.climateengine.org/).

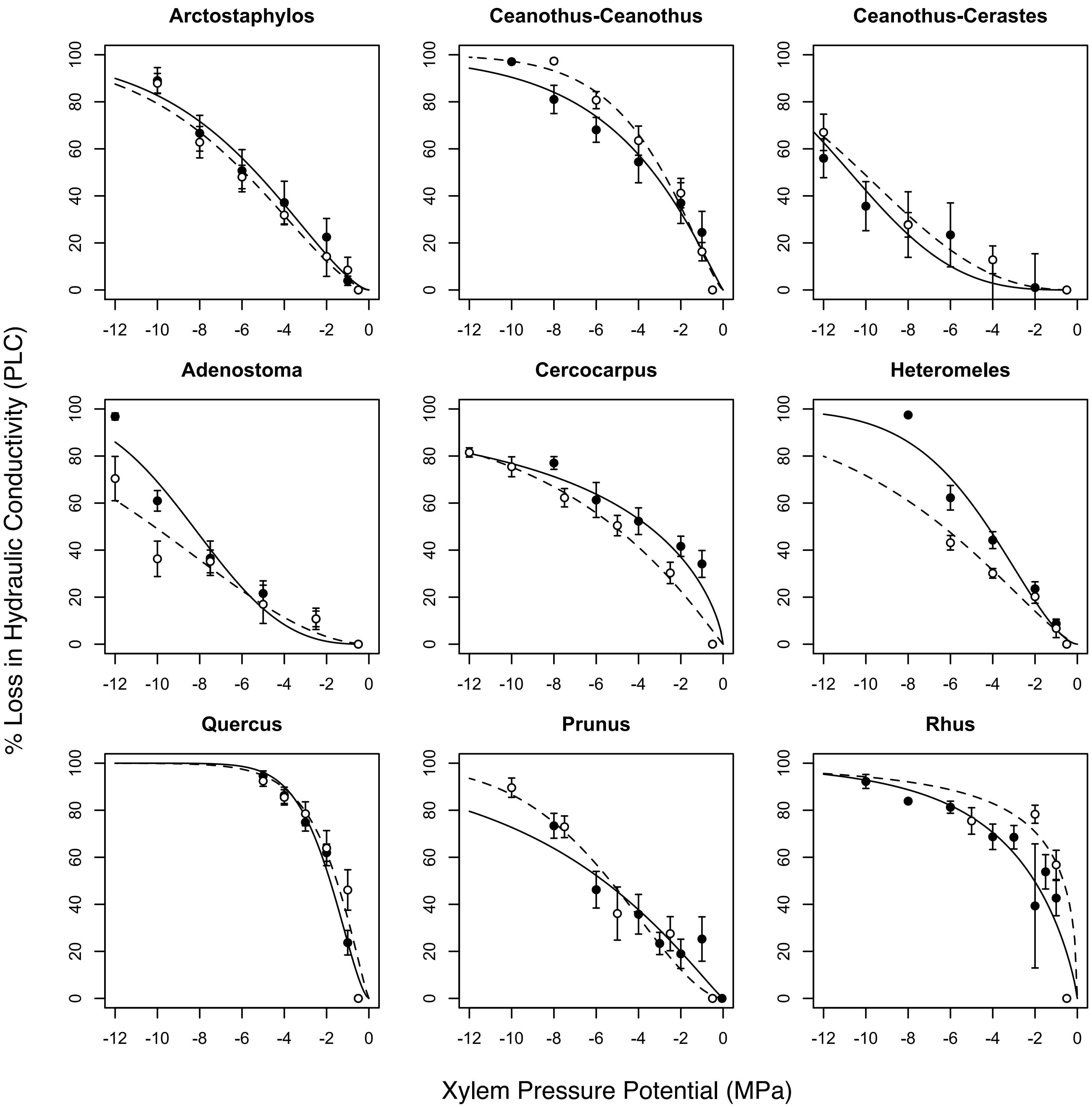

Figure S2a. Vulnerability to cavitation curves for 9 congeneric species pairs generated using a standard centrifuge technique (Alder et al. 1997; Sperry et al. 1988; Tobin et al. 2013). Curves show percentage loss in hydraulic conductivity (PLC; y-axis) vs. xylem pressure potential (x-axis). Points are means based on data collected for *n* = 6 stems per species. Regression lines represent a 2-parameter Weibull function fit to mean data for each species. Curves were used to calculate the xylem pressure at 50% loss in conductivity (Ψ_{50}).

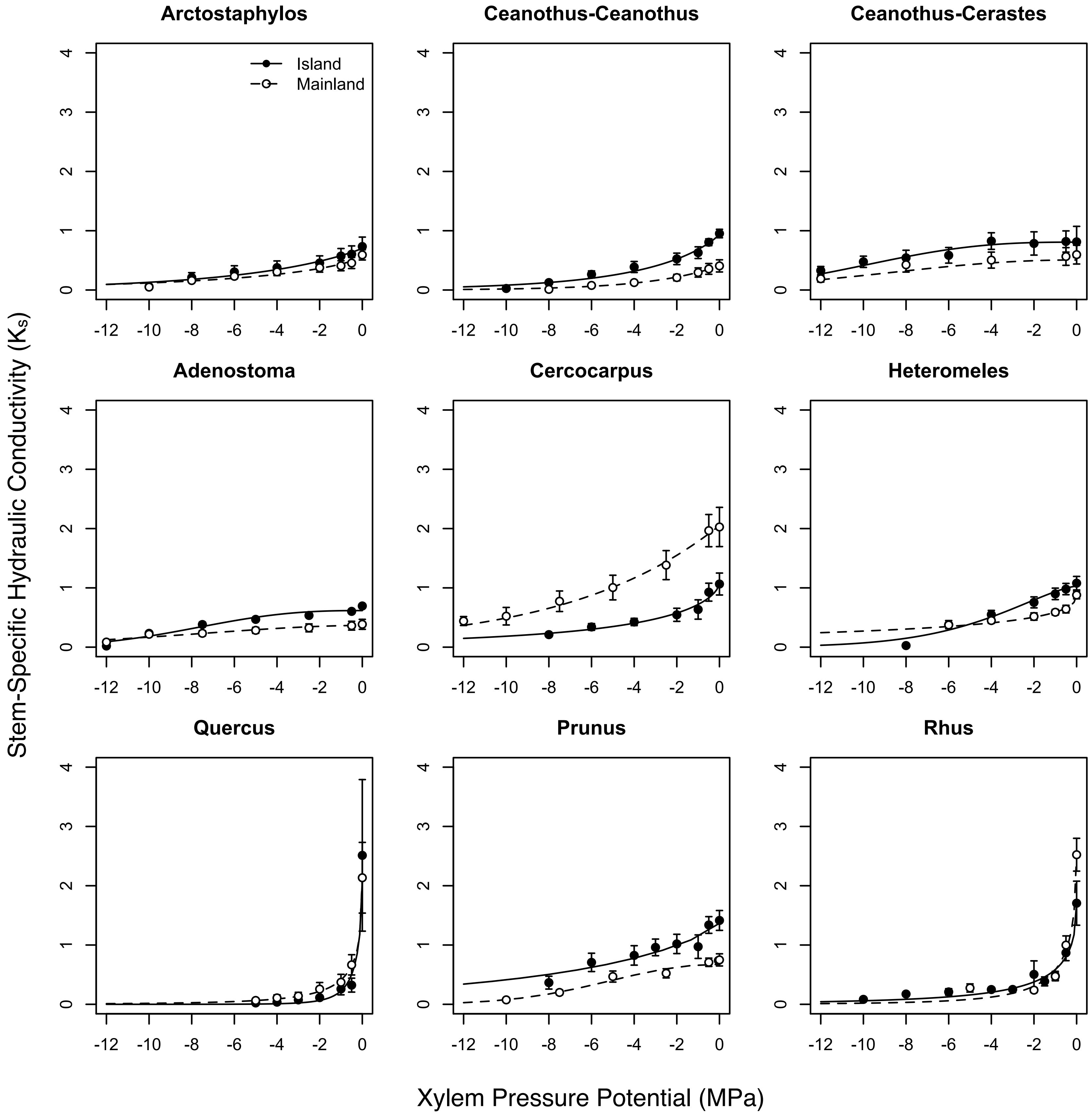

Figure S2b. Hydraulic conductivity curves for 9 congeneric species pairs generated using a standard centrifuge technique (Alder et al. 1997; Sperry et al. 1988; Tobin et al. 2013). Curves show stem-specific hydraulic conductivity (K_s; y-axis) vs. xylem pressure potential (x-axis). Each curve is based on data collected for n = 6 stems per species. Regression lines represent a 2-parameter Weibull function fit to mean data for each species.

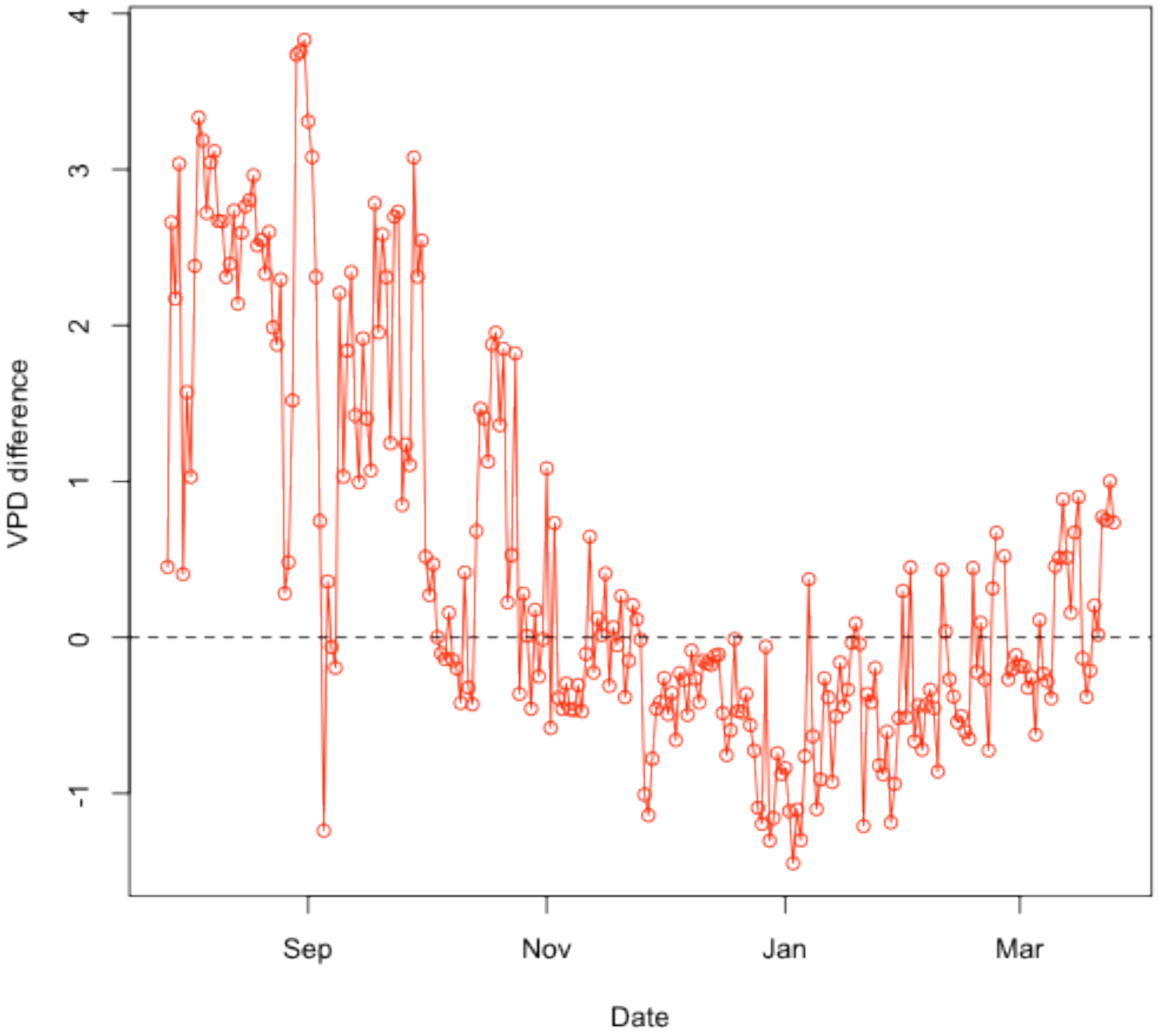

Figure S3. Difference in daily max. vapor pressure deficit (VPD) between island (Santa Catalina Island) and mainland (Santa Ana Mountains) sites. Positive VPD difference values indicate that mainland VPD is higher than island VPD. This suggests that on average mainland plants experience higher evaporative demand during the summer, dry-season. VPD calculated from temperature and relative humidity measured hourly using HOBO temp/rH dataloggers (ONSET, Pro V2, Bourne, MA, USA).

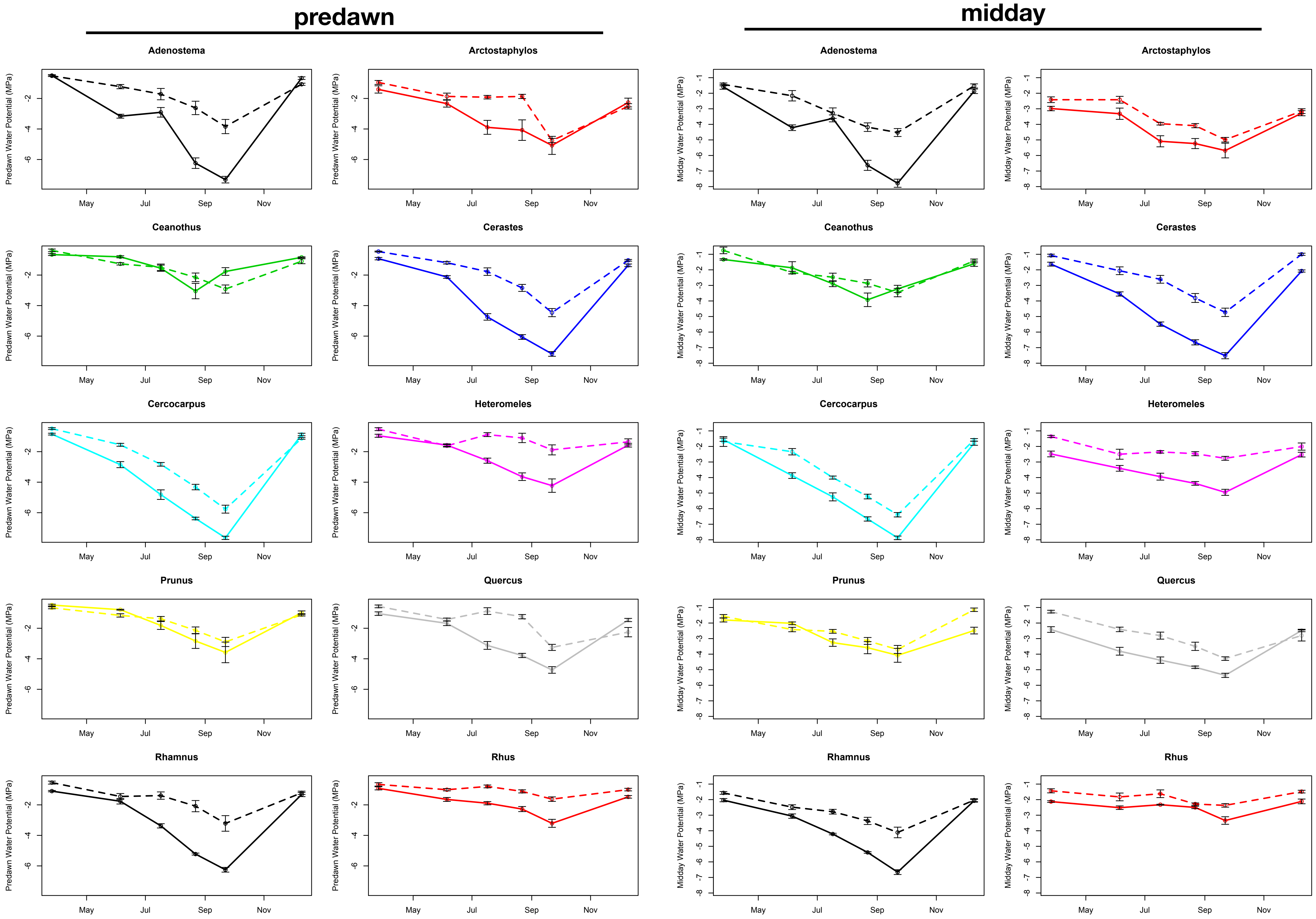

Figure S4. Mean predawn (Ψ_{pd}) and midday (Ψ_{md}) water potential between island (Santa Catalina Island; dashed lines) and mainland (Santa Ana Mountains; solid lines) sites. Taxonomic pairs are indicated by figure titles and coloring across datasests.

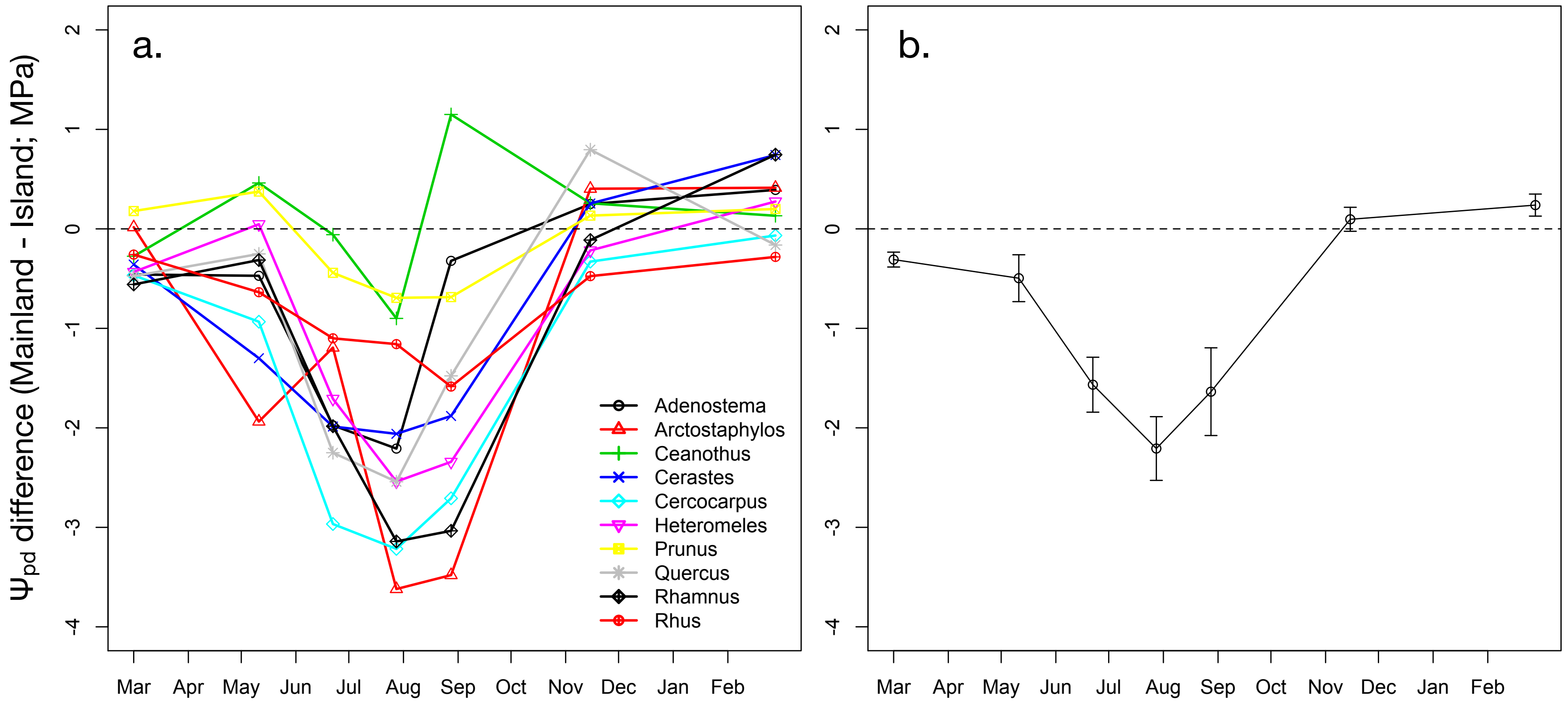

Figure S5. Difference in predawn water potential (Ψ_{pd}) between island (Santa Catalina Island) and mainland (Santa Ana Mountains) sites for each species pair (a.) and averaged for all pairs (b.). Negative difference values indicate that mainland Ψ_{pd} is more negative than island Ψ_{pd}. This suggests that on average mainland plants experience more dehydrated leaf tissues, especially during the dry season—an average difference of > 2 Mpa.

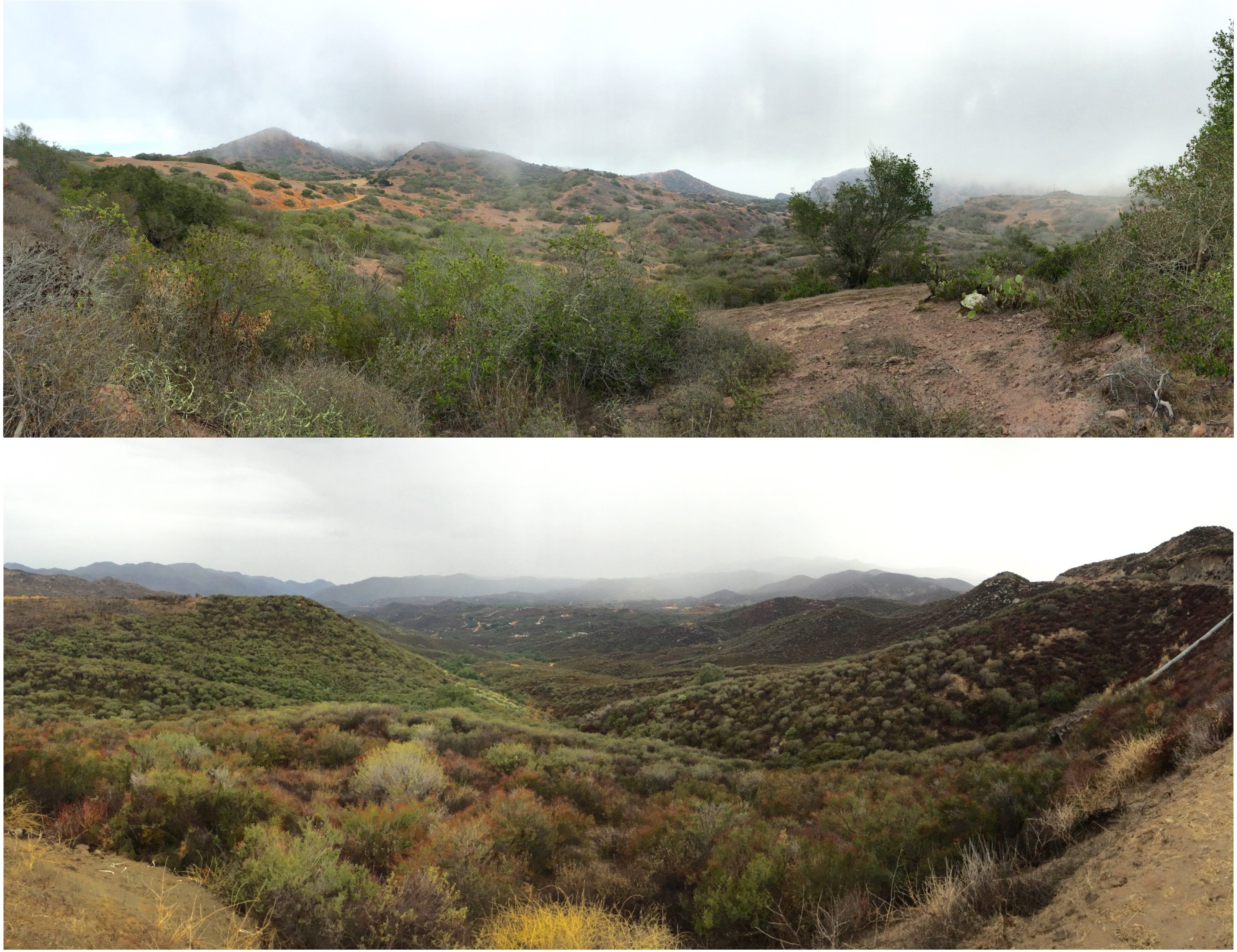

Figure S6. Photographs of chaparral field sites from Santa Catalina Island (a) and the Santa Ana Mountains (b). These photographs illustrate the differences between typical island and mainland chaparral communities with the island exhibiting a more open canopy structure with greater spacing between fewer individuals.

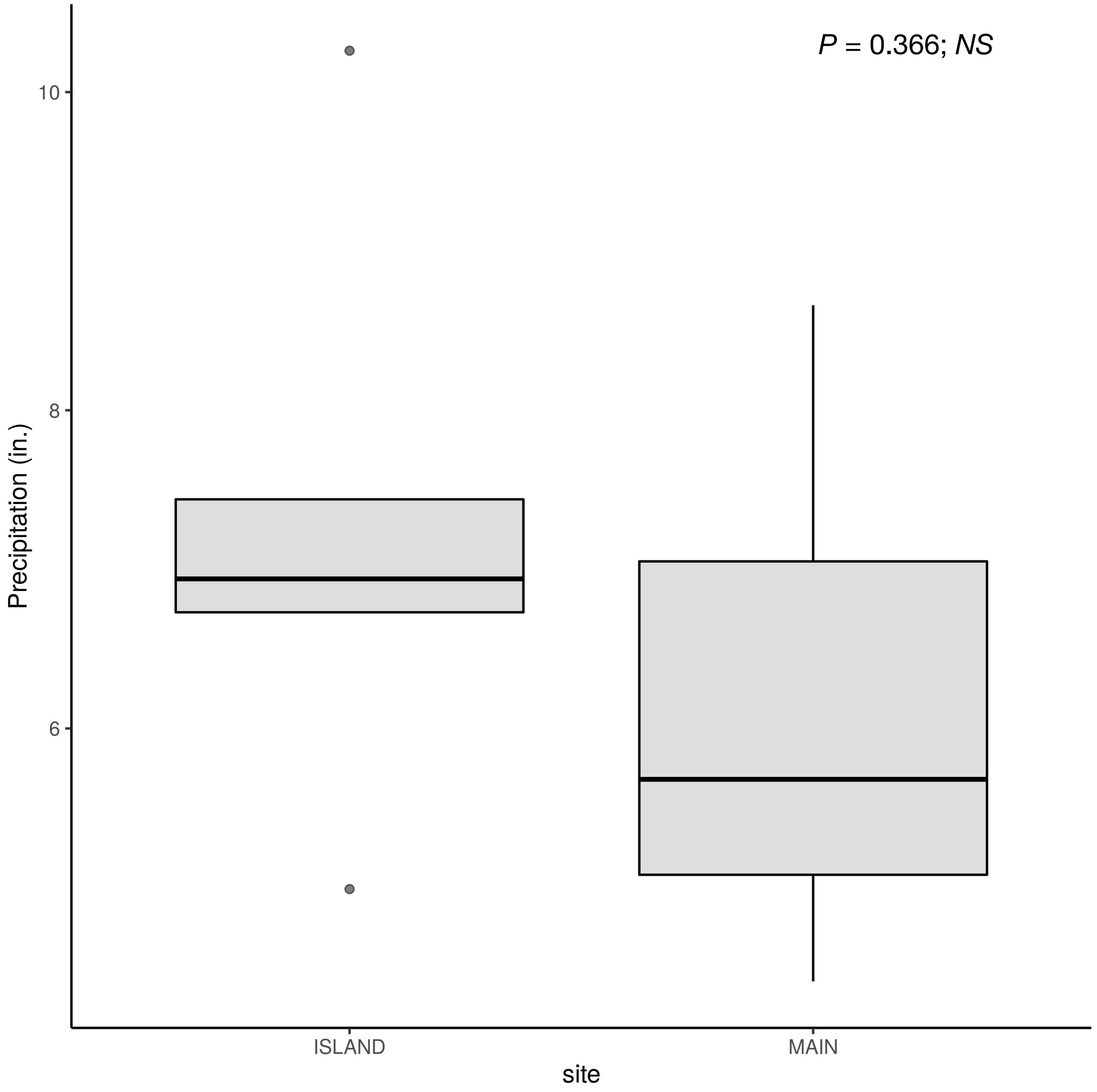

Figure S7. Mean Annual Precipitation during the recent multi-year drought (2012-2016) from island (Santa Catalina Island) and mainland (Santa Ana Mountains) field locations. MAP is not different between sites ($t_{7.89}$ = 0.96; *p-value* = 0.366), suggesting similar precipitation anomalies during this historic dry period.

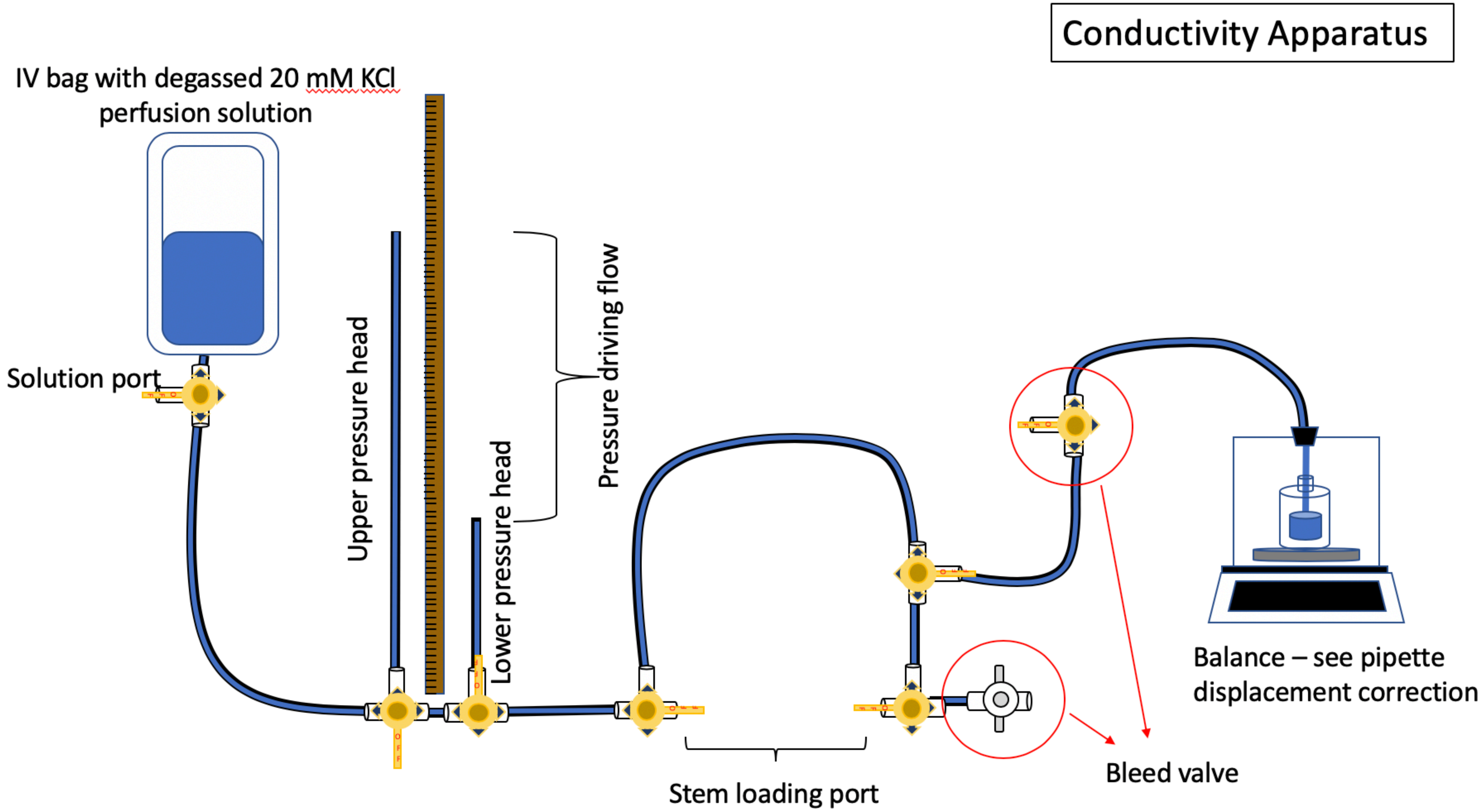

Figure S8. Schematic of conductivity apparatus used to measure stem-specific hydraulic conductivity. System was constructed with Bev-a-line tubing (ID 1/8", OD 1/4"; Cole-Parmer, Vernon Hills, IL) and Leurlock valves (Cole-Parmer, Vernon Hills, IL), a meter stick, ring stands (to support tubing and ruler). A 4-digit balance (Sartorius, Practum 224, Goettingen, Germany) was connected to a computer via USB.

Table S1. Summary table of repeated-measures ANOVAs for predawn water potential (Ψ_{pd}), midday water potential (Ψ_{md}), and stomatal conductance (g_s).

Factor	df	Ψ_{pd}	Ψ_{md}	g_s
site	*1*	**257.21*** **	**394.60*** **	**12.17*** **
genus	*10*	**33.26*** **	**51.55*** **	**11.21*** **
date	*6*	**2.90* **	1.17	0.78
site x genus	*9*	**6.93*** **	**8.00*** **	**10.81*** **
site x date	*3*	0.63	0.06	0.04
genus x date	*5*	0.26	0.79	3.05
residuals df		*91*	*96*	*97*

Table S2. Trait means (±1SE) for stem hydraulic traits and seasonal water relations from 10 congeneric island-mainland pairs. ANOVA results for the main factor (genus within site) for each trait are reported at the bottom of each column. Bold p-values indicate statistically significant effects according to mixed model ANOVAs ($P < 0.05$). Bold mean values within the table indicate statistically significant pairwise comparisons ($P < 0.05$).

			Hydraulics Traits			Seasonal Water Relations				Safety Margin
Family	Species	Site	P_{50} (MPa)	k_s (m^2 Mpa^{-1} s^{-1} 10^3)	XD (g cm^3)	Ψ_{max} (MPa)	Ψ_{min} (MPa)	g_{smax} (mmol m^{-2} s^{-1})	ϕ_{PSII} (F_v / F_m)	$Safety_{P50}$ (MPa)
Anacardiaceae	*Rhus integrifolia*	SCI	-1.908 ± 0.340	1.705 ± 0.372	0.545 ± 0.020	**-0.517 ± 0.071**	**-2.477 ± 0.067**	**161.820 ± 44.515**	0.719 ± 0.016	**-0.793 ± 0.114**
	Rhus ovata	SAM	-1.345 ± 0.202	2.523 ± 0.277	0.573 ± 0.011	**-0.767 ± 0.081**	**-3.350 ± 0.239**	**26.383 ± 11.514**	0.654 ± 0.035	**-2.266 ± 0.242**
Ericaceae	*Arctostaphylos catalinae*	SCI	-5.998 ± 0.973	0.733 ± 0.159	0.647 ± 0.016	-0.504 ± 0.038	**-4.153 ± 0.435**	54.200 ± 29.223	**0.722 ± 0.020**	**0.699 ± 0.279**
	Arctostaphylos glauca	SAM	-5.885 ± 0.590	0.592 ± 0.086	0.644 ± 0.011	-0.470 ± 0.042	**-7.833 ± 0.230**	4.133 ± 0.886	**0.350 ± 0.044**	**-2.006 ± 0.261**
Fagaceae	*Quercus pacifica*	SCI	-1.848 ± 0.123	2.512 ± 1.278	0.694 ± 0.014	-0.233 ± 0.319	**-4.285 ± 0.116**	**246.200 ± 40.072**	0.798 ± 0.009	**-2.303 ± 0.116**
	Quercus berberidifolia	SAM	-1.598 ± 0.205	2.135 ± 0.596	0.741 ± 0.016	-0.825 ± 0.083	**-5.362 ± 0.135**	**94.858 ± 37.662**	0.708 ± 0.038	**-4.098 ± 0.135**
Rhamnaceae	*Ceanothus arboreus*	SCI	-3.378 ± 0.602	**0.953 ± 0.071**	0.695 ± 0.017	-0.383 ± 0.089	-3.462 ± 0.269	172.467 ± 25.705	0.821 ± 0.007	-0.406 ± 0.269
	Ceanothus oliganthus	SAM	-2.905 ± 0.312	**0.407 ± 0.103**	0.660 ± 0.014	-0.603 ± 0.058	-3.982 ± 0.427	317.667 ± 100.133	0.806 ± 0.009	-1.156 ± 0.435
	Ceanothus megacarpus var. *insularis*	SCI	-9.638 ± 1.259	0.812 ± 0.262	0.722 ± 0.017	**-0.492 ± 0.060**	**-5.867 ± 0.538**	**66.840 ± 12.922**	**0.732 ± 0.013**	**5.006 ± 0.155**
	Ceanothus crassifolius	SAM	-10.20 ± 0.945	0.519 ± 0.150	0.698 ± 0.014	**-0.746 ± 0.060**	**-7.875 ± 0.116**	**27.690 ± 4.542**	**0.568 ± 0.034**	**2.292 ± 0.116**
	Rhamnus pirifolia	SCI	*NA*	0.600 ± 0.147	0.655 ± 0.009	**-0.542 ± 0.098**	**-4.108 ± 0.340**	96.180 ± 15.814	**0.747 ± 0.020**	*NA*
	Rhamnus ilicifolia	SAM	*NA*	0.883 ± 0.153	0.665 ± 0.014	**-0.946 ± 0.079**	**-6.658 ± 0.154**	126.717 ± 16.778	**0.625 ± 0.033**	*NA*
Rosaceae	*Adenostoma fasciculatum*	SCI	-8.252 ± 0.490	**0.695 ± 0.020**	0.690 ± 0.030	-0.908 ± 0.096	-4.988 ± 0.149	**45.317 ± 9.783**	**0.780 ± 0.014**	**3.295 ± 0.149**
		SAM	-9.852 ± 0.840	**0.385 ± 0.084**	0.748 ± 0.018	-1.125 ± 0.163	-5.817 ± 0.394	**9.733 ± 3.754**	**0.692 ± 0.022**	**5.216 ± 0.471**
	Cercocarpus betuloides	SCI	-3.733 ± 0.679	**1.065 ± 0.185**	0.727 ± 0.024	**-0.458 ± 0.030**	**-4.730 ± 0.268**	84.283 ± 7.674	**0.832 ± 0.004**	**-1.631 ± 0.268**
		SAM	-5.218 ± 0.623	**2.027 ± 0.331**	0.693 ± 0.060	**-0.900 ± 0.067**	**-7.517 ± 0.198**	55.933 ± 11.771	**0.631 ± 0.036**	**-2.613 ± 0.198**
	Heteromeles arbutifolia	SCI	**-4.295 ± 0.278**	1.079 ± 0.114	0.633 ± 0.022	**-0.525 ± 0.095**	**-3.050 ± 0.180**	**266.917 ± 24.772**	0.755 ± 0.025	1.426 ± 0.125
		SAM	**-6.906 ± 0.383**	0.880 ± 0.065	0.682 ± 0.022	**-0.854 ± 0.084**	**-4.950 ± 0.195**	**75.092 ± 35.265**	0.609 ± 0.068	1.033 ± 0.195
	Prunus ilicifolia ssp. *lyonii*	SCI	-5.633 ± 0.824	**1.415 ± 0.167**	0.664 ± 0.019	-0.650 ± 0.063	-3.713 ± 0.245	161.750 ± 17.275	**0.757 ± 0.021**	**1.803 ± 0.258**
	Prunus ilicifolia ssp. *ilicifolia*	SAM	-5.454 ± 0.756	**0.793 ± 0.083**	0.632 ± 0.016	-0.471 ± 0.055	-4.275 ± 0.349	131.800 ± 34.109	**0.624 ± 0.052**	**0.526 ± 0.420**
	ANOVA Results	F	3.952	0.059	0.405	25.379	117.160	8.240	72.235	37.950
		df	1, 94	1, 107	1, 109	1, 109	1, 109	1, 104	1, 104	1, 94
		P	0.117	0.809	0.526	**< 0.001**	**< 0.001**	**< 0.01**	**< 0.001**	**< 0.001**

Table S3. Summary table of mixed model ANOVA results for cavitation resistance (P_{50}) and hydraulic safety margins (HSM_{50}). Analyses performed on all data, r-shaped curves only, and all other curves (s and exp.-shaped). Bold text indicates statistically significant *p*-values at an α of 0.016 (Bonferroni-adjusted *p*-value to account for increased Type I error rate with multiple tests). Findings of no difference in P_{50} and highly significant difference (**bold text**) in HSM_{50} are consistent across all analytical approaches.

site(genus)	P_{50} all data	P_{50} r-shaped	P_{50} other	HSM all data	HSM r-shaped	HSM other
F	2.502	0.023	4.754	37.950	49.440	10.890
df	*1, 95*	*1, 43*	*1, 52*	*1, 95*	*1, 43*	*1, 52*
P	0.117	0.879	0.034	**< 0.001**	**< 0.001**	**0.002**
